# Supplementary material for: PilT and PilU are homohexameric ATPases that coordinate to retract type IVa pili
Source: PLoS Genet. 2019 Oct 18;15(10):e1008448. doi: 10.1371/journal.pgen.1008448 (PMC6821130; doi:10.1371/journal.pgen.1008448)
Supplement: S1 Text — (PDF) [file pgen.1008448.s001.pdf]

# Supporting Information for:

## PilT and PilU are homohexameric ATPases that coordinate to retract type IVa pili

Jennifer L. Chlebek[1], Hannah Q. Hughes[1], Aleksandra S. Ratkiewicz[2], Rasman Rayyan[2], Joseph Che-Yen Wang[3], Brittany E. Herrin[1], Triana N. Dalia[1], Nicolas Biais[2], and Ankur B. Dalia[1,*]

[1]Department of Biology, Indiana University, Bloomington, Indiana, United States of America

[2]Biology Department and Graduate Center, City University of New York, Brooklyn, New York, United States of America

[3]Electron Microscopy Center, Indiana University, Bloomington, Indiana, United States of America

*Correspondence to: ankdalia@indiana.edu.

**This PDF file includes:**

S1-S5 Figs.
S1-S4 Tables
