# Supplementary figures and images for: PilT and PilU are homohexameric ATPases that coordinate to retract type IVa pili
Source: PLoS Genet. 2019 Oct 18;15(10):e1008448. doi: 10.1371/journal.pgen.1008448 (PMC6821130; doi:10.1371/journal.pgen.1008448)

Transformation frequency  
(transformants/totalCFU)

$P_{tac}$ -*pilT*

$P_{tac}$ -*pilU*

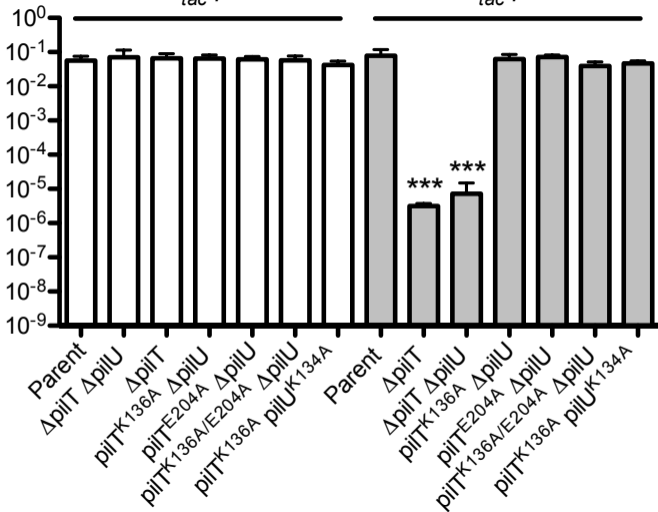

Supplement: S1 Fig — Natural transformation assays of the indicated strains. All strains contain either a chromosomally integrated IPTG-inducible Ptac-pilT (white bars) or Ptac-pilU (gray bars) construct as indicated. Data show that ectopic overexpression of PilT and PilU does not affect the transformation frequency of the parent strain. Ectopic overexpression of PilT rescued the transformation of all strains that showed a substantial reduction in natural transformation in Fig 1A and Fig 2B (i.e. ΔpilT, ΔpilT ΔpilU, pilTK136A ΔpilU, pilTE204A ΔpilU, pilTE204A/K136A ΔpilU, and pilTK136A pilUK134A). Ectopic overexpression of PilU rescued all strains except for strains that lacked a copy of pilT (i.e. ΔpilT, ΔpilT ΔpilU). All strains were grown with 100 μM IPTG to ectopically induce PilT or PilU. Data are shown as the mean ± SD. Parent, n = 8. All other strains, n = 4. Asterisk(s) directly above bars denote comparisons to the appropriate parent strain. Comparisons were made by grouping data into the two families indicated by the white and grey bars. A one-way ANOVA was performed for each family with a Dunnet’s post to compare each experimental sample to the parent strain. Comparisons were not statistically significantly different unless otherwise noted. *** = P < 0.001. (PDF) [file pgen.1008448.s002.pdf]

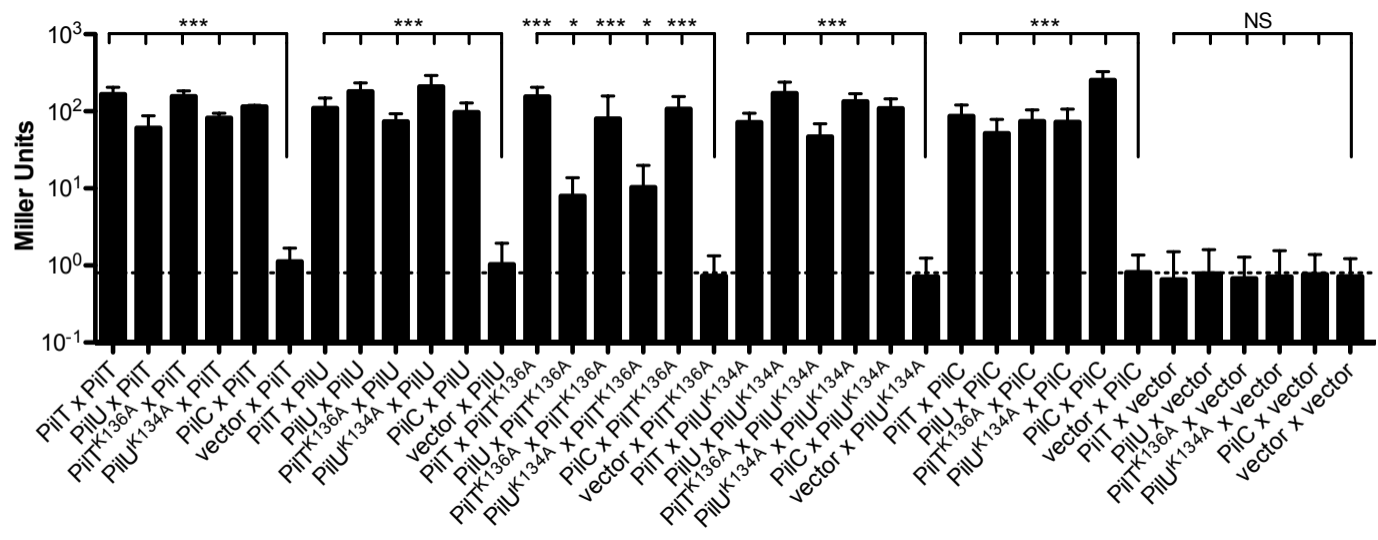

Supplement: S2 Fig — BACTH Miller assays showing all data from Fig 3B with additional negative controls for every T25- or T18-fusion. Data are shown as the mean ± SD from three independent experiments. The dotted line represents the β-galactosidase activity of the empty vector negative control (T18-empty x T25-empty). Comparisons were made by grouping data into appropriate families indicated by the black lines above the bar graph. A one-way ANOVA was performed on each family with a Dunnet’s post test to compare each experimental group to the appropriate negative control (indicated by the longer black line). A single set of asterisks directly above the group line denotes the statistical results for all comparisons in that family unless otherwise noted. NS, not significant; * = P < 0.05; *** = P < 0.001. (PDF) [file pgen.1008448.s003.pdf]

**A**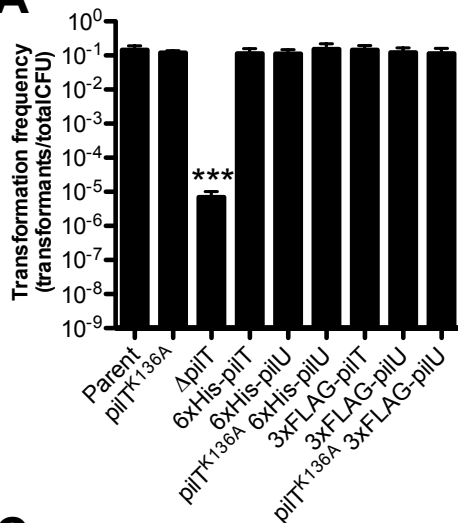**B**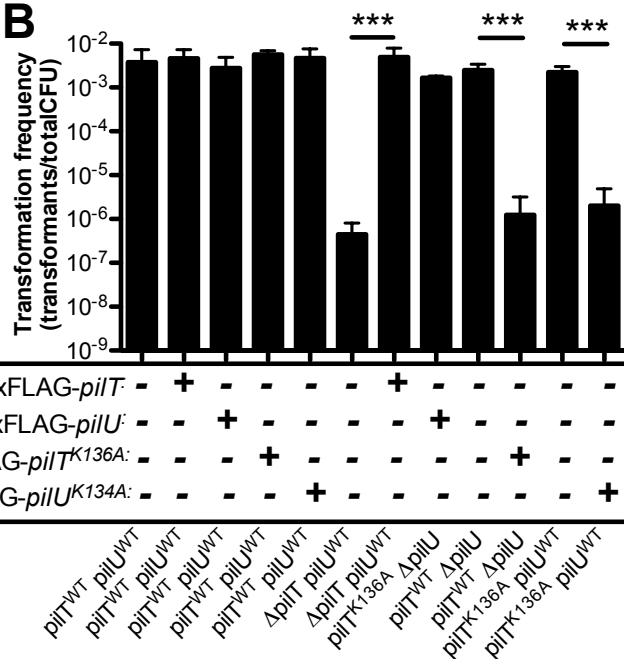**C**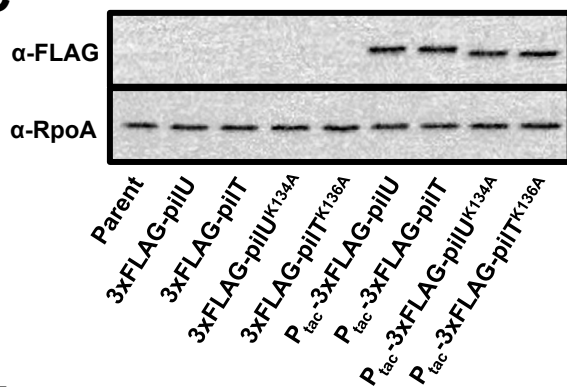**D**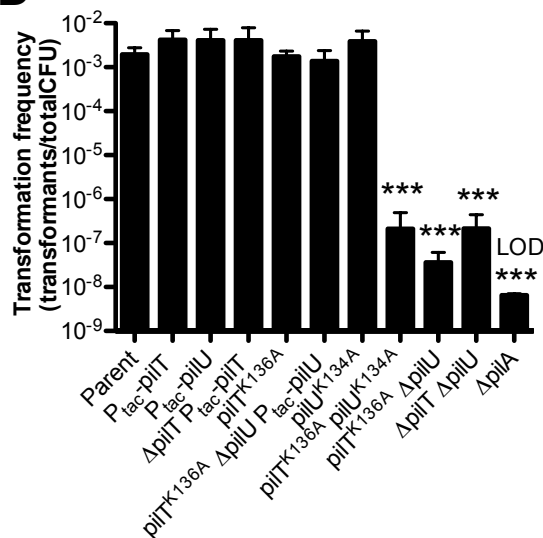**E**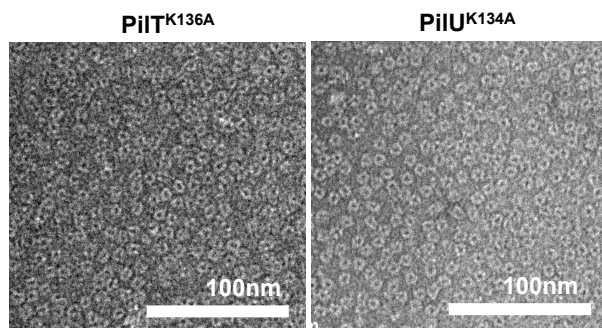

Supplement: S3 Fig — (A) Natural transformation assays showing that 6xHis and 3xFLAG N-terminal fusions to PilT and PilU are functional. The indicated strains were incubated with 500 ng of transforming DNA overnight. Parent, n = 8. All others, n = 4. (B) Natural transformation assays showing that overexpression of 3xFLAG tagged PilT/PilU/PilTK136A/PilUK134A behave the same as non-tagged proteins when ectopically overexpressed in the indicated backgrounds. Strains were incubated with 500 ng of transforming DNA and after 7 minutes, DNAse I was added to prevent additional DNA uptake. Parent, n = 5. pilTK136A, n = 5. All others, n = 4. (C) Western blot of the indicated strains to detect FLAG tagged-proteins and RpoA as a loading control. Strains containing an IPTG regulated Ptac constuct were grown in the presence of 100 μM IPTG. This blot indicates that ectopic induction of 3xFLAG tagged PilT/PilU/PilTK136A/PilUK134A results in robust overexpression of proteins above native levels. Data is representative of three independent experiments. (D) Natural transformation assays where the indicated strains were incubated with 500 ng of transforming DNA for 7 minutes prior to the addition of DNAse I to prevent additional DNA uptake. These data indicate that ectopic overexpression of PilT or PilU can rescue strains with a substantial reduction in transformation frequency. All strains with Ptac constructs were grown with 100 μM IPTG to overexpress PilT or PilU. All bar graphs are shown as the mean ± SD. Asterisk(s) directly above bars denote comparisons to parent strain. All comparisons were made by one-way ANOVA followed with Tukey’s post test. LOD, limit of detection; *** = P < 0.001. (E) Representative negative stain transmission electron micrographs showing that purified 6XHis-PilTK136A and 6XHis-PilUK134A form hexamers in vitro. Scale bar, 100 nm. (PDF) [file pgen.1008448.s004.pdf]

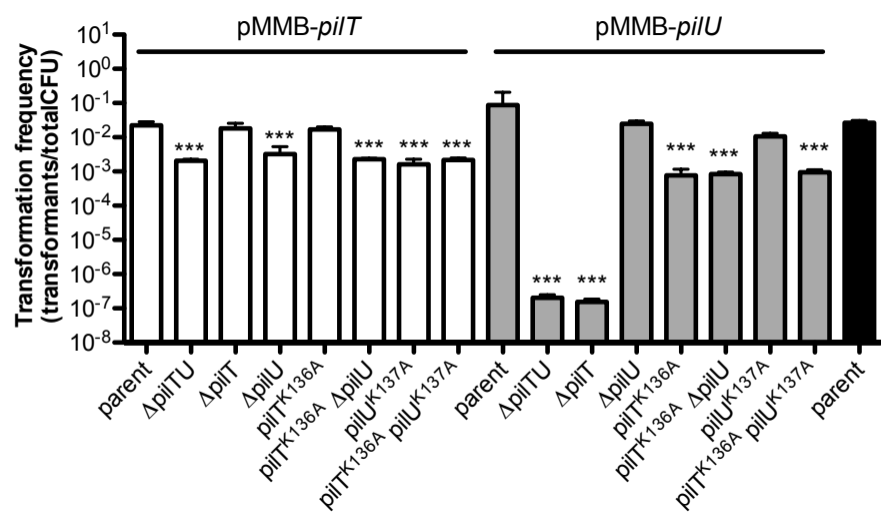

Supplement: S4 Fig — Natural transformation assays of the indicated A. baylyi strains. Strains harbored pMMB-pilT (white bars), pMMB-pilU (gray bars), or no vector (black bar). Data indicate that ectopic overexpression of PilT and PilU do not affect the transformation frequency of the parent strain. Ectopic overexpression of PilT rescued the transformation of all strains that showed a significant reduction in natural transformation in Fig 5 (i.e. ΔpilT, ΔpilT ΔpilU, pilTK136A ΔpilU, and pilTK136A pilUK137A). Ectopic overexpression of PilU rescued all strains except for strains that lacked a copy of pilT (i.e. ΔpilT and ΔpilT ΔpilU). All strains were grown with 100 μM IPTG to induce expression of pilT or pilU. Data are shown as the mean ± SD and are from three independent experiments. Asterisk(s) directly above bars denote comparisons to the appropriate parent strain. Comparisons were made by grouping data into the two families indicated by the white and grey bars. A one-way ANOVA was performed for each family followed by Dunnet’s post test to compare each experimental group to the parent strain. Comparisons were not statistically significantly different from the relevant parent unless otherwise noted. *** = P < 0.001. (PDF) [file pgen.1008448.s005.pdf]

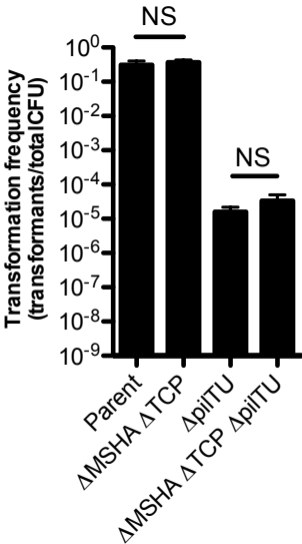

Supplement: S5 Fig — Natural transformation assays of the indicated strains. Reactions were incubated with 500 ng of transforming DNA overnight. ΔMSHA and ΔTCP represent deletions of the entire locus for both pilus systems, which includes the extension ATPase associated with each. Data are shown as the mean ± SD and are from four independent biological replicates. All comparisons were made by one-way ANOVA with Tukey’s post test. NS, not significant. (PDF) [file pgen.1008448.s006.pdf]
