# Supplementary material for: PilT and PilU are homohexameric ATPases that coordinate to retract type IVa pili
Source: PLoS Genet. 2019 Oct 18;15(10):e1008448. doi: 10.1371/journal.pgen.1008448 (PMC6821130; doi:10.1371/journal.pgen.1008448)
Supplement: S1 Table — (PDF) [file pgen.1008448.s007.pdf]

S1 Table. Strains used in this study

Strain name in manuscript	Genotype and antibiotic resistances	Figure	Strain #
Parent	E7946 SmR, ΔlacZ::lacIq, P_{tac}-tfoX, ΔluxO::miniFRT, ΔVC1807::ZeoR, pilA S56C	Fig 1, Fig 2	TND0905 (SAD2468)
ΔpilU	E7946 SmR, ΔlacZ::lacIq, P_{tac}-tfoX, ΔluxO::SpecR, ΔVC1807::CmR, pilA S56C, comEA-mCherry, ΔpilU::TmR *	Fig 1, Fig 2	SAD2135
ΔpilT	E7946 SmR, ΔlacZ::lacIq, P_{tac}-tfoX, ΔluxO::miniFRT, ΔVC1807::ZeoR, pilA S56C, ΔpilT::TmR	Fig 1, Supp Fig 3	TND1035 (SAD2469)
ΔpilTU	E7946 SmR, ΔlacZ::lacIq, P_{tac}-tfoX, ΔluxO::miniFRT, ΔVC1807::ZeoR, pilA S56C, ΔpilTU::TmR	Fig 1	JLC227 (SAD2470)
pilT^{K136A}	E7946 SmR, ΔlacZ::lacIq, P_{tac}-tfoX, ΔluxO::miniFRT, ΔVC1807::KanR, pilA S56C, pilT ^{K136A}	Fig 1, Fig 2	TND1077 (SAD2471)
pilT^{K136A} ΔpilU	E7946 SmR, ΔlacZ::lacIq, P_{tac}-tfoX, ΔluxO::miniFRT, ΔVC1807::ZeoR, pilA S56C, pilT ^{K136A}, ΔpilU::TmR	Fig 1	JLC444 (SAD2472)
pilT^{E204A}	E7946 SmR, ΔlacZ::lacIq, P_{tac}-tfoX, ΔluxO::miniFRT, ΔVC1807::CmR, pilA S56C, pilT ^{E204A}	Fig 1	TND1203 (SAD2473)
pilT$^{E204A/K136A}$	E7946 SmR, ΔlacZ::lacIq, P_{tac}-tfoX, ΔluxO::miniFRT, ΔVC1807::CmR, pilA S56C, pilT ^{E204A}/^{K136A}	Fig 1	JLC324 (SAD2474)
pilT$^{E204A/K136A}$ ΔpilU	E7946 SmR, ΔlacZ::lacIq, P_{tac}-tfoX, ΔluxO::miniFRT, ΔVC1807::CmR, pilA S56C, pilT ^{E204A}/^{K136A}, ΔpilU::TmR	Fig 1	JLC530 (SAD2475)
pilT^{E204A} ΔpilU	E7946 SmR, ΔlacZ::lacIq, P_{tac}-tfoX, ΔluxO::miniFRT, ΔVC1807::CmR, pilA S56C, pilT ^{E204A}, ΔpilU::TmR	Fig 1	JLC531 (SAD2476)
ΔpilA	E7946 SmR, ΔlacZ::lacIq, P_{tac}-tfoX, ΔluxO::SpecR, ΔVC1807::KanR, ΔpilA::miniFRT	Fig 1	TND0855 (SAD2477)
Parent (micropillar assays)	E7946 SmR, ΔlacZ::lacIq, P_{tac}-tfoX, ΔluxO::SpecR, ΔVC1807::CmR, pilA S56C, ΔflaA::CarbR, ΔmshA::miniFRT, Δvps-rbmA::ZeoR, ΔtcpA::KanR	Fig 1	TND0752 (SAD2478)
ΔpilT (micropillar assays)	E7946 SmR, ΔlacZ::lacIq, P_{tac}-tfoX, ΔluxO::SpecR, ΔVC1807::CmR, pilA S56C, ΔflaA::CarbR, ΔmshA::miniFRT, Δvps-rbmA::ZeoR, ΔtcpA::KanR, ΔpilT::TmR	Fig 1	TND0767 (SAD2479)
ΔpilU (micropillar assays)	E7946 SmR, ΔlacZ::lacIq, P_{tac}-tfoX, ΔluxO::SpecR, ΔVC1807::CmR, pilA S56C, ΔflaA::CarbR, ΔmshA::miniFRT, Δvps-rbmA::ZeoR, ΔtcpA::KanR, ΔpilU::TmR	Fig 1	TND1168 (SAD2480)
ΔpilT ΔpilU (micropillar assays)	E7946 SmR, ΔlacZ::lacIq, P_{tac}-tfoX, ΔluxO::SpecR, ΔVC1807::CmR, pilA S56C,	Fig 1	TND1174 (SAD2635)

	ΔflaA::CarbR, ΔmshA::miniFRT, Δvps-rbmA::ZeoR, ΔtcpA::KanR, ΔpilTU::TmR		
P_{tac}-PilT	E7946 SmR, P_{tac}-tfoX, ΔluxO::miniFRT, ΔVC1807::ZeoR, pilA S56C, ΔlacZ::P_{tac}-PilT SpecR	Supp Fig 1	JLC426 (SAD2481)
P_{tac}-PilU	E7946 SmR, P_{tac}-tfoX, ΔluxO::miniFRT, ΔVC1807::ZeoR, pilA S56C, ΔlacZ::P_{tac}-PilU SpecR	Supp Fig 1	JLC427 (SAD2482)
P_{tac}-PilT ΔpilTU	E7946 SmR, P_{tac}-tfoX, ΔluxO::miniFRT, ΔVC1807::ZeoR, pilA S56C, ΔlacZ::P_{tac}-PilT SpecR, ΔpilTU::TmR	Supp Fig 1	JLC420 (SAD2483)
P_{tac}-PilU ΔpilTU	E7946 SmR, P_{tac}-tfoX, ΔluxO::miniFRT, ΔVC1807::ZeoR, pilA S56C, ΔlacZ::P_{tac}-PilU SpecR, ΔpilTU::TmR	Supp Fig 1	JLC421 (SAD2484)
P_{tac}-PilT pilT^{K136A} ΔpilU	E7946 SmR, P_{tac}-tfoX, ΔluxO::miniFRT, ΔVC1807::KanR, pilA S56C, ΔlacZ::P_{tac}-PilT SpecR, pilT ^{K136A}, ΔpilU::TmR	Supp Fig 1	JLC450 (SAD2485)
P_{tac} -PilU pilT^{K136A} ΔpilU	E7946 SmR, P_{tac}-tfoX, ΔluxO::miniFRT, ΔVC1807::KanR, pilA S56C, ΔlacZ::P_{tac}-PilU SpecR, pilT ^{K136A}, ΔpilU::TmR	Supp Fig 1	JLC451 (SAD2486)
P_{tac} -PilT pilT^{K136A} pilU^{K134A}	E7946 SmR, P_{tac}-tfoX, ΔluxO::miniFRT, ΔVC1807::KanR, pilA S56C, ΔlacZ::P_{tac}-PilT SpecR, pilT ^{K136A} pilU ^{K134A}	Supp Fig 1	JLC424 (SAD2487)
P_{tac}-PilU pilT^{K136A} pilU^{K134A}	E7946 SmR, P_{tac}-tfoX, ΔluxO::miniFRT, ΔVC1807::KanR, pilA S56C, ΔlacZ::P_{tac}-PilU SpecR, pilT ^{K136A} pilU ^{K134A}	Supp Fig 1	JLC425 (SAD2488)
P_{tac}-PilT ΔpilTU	E7946 SmR, P_{tac}-tfoX, ΔluxO::miniFRT, ΔVC1807::ZeoR, pilA S56C, ΔlacZ::P_{tac}-PilT SpecR, ΔpilTU::TmR	Supp Fig 1	JLC438 (SAD2489)
P_{tac}-PilU ΔpilTU	E7946 SmR, P_{tac}-tfoX, ΔluxO::miniFRT, ΔVC1807::ZeoR, pilA S56C, ΔlacZ::P_{tac}-PilU SpecR, ΔpilTU::TmR	Supp Fig 1	JLC439 (SAD2490)
P_{tac}-PilT pilT$^{E204A/K136A}$ ΔpilU	E7946 SmR, P_{tac}-tfoX, ΔluxO::miniFRT, ΔVC1807::CmR, pilA S56C, pilT ^{E204A}/^{K136A}, ΔpilU::TmR, ΔlacZ::P_{tac}-PilT SpecR	Supp Fig 1	JLC540 (SAD2491)
P_{tac}-PilU pilT$^{E204A/K136A}$ ΔpilU	E7946 SmR, P_{tac}-tfoX, ΔluxO::miniFRT, ΔVC1807::CmR, pilA S56C, pilT ^{E204A}/^{K136A}, ΔpilU::TmR, ΔlacZ::P_{tac}-PilU SpecR	Supp Fig 1	JLC541 (SAD2492)
P_{tac}-PilT pilT^{E204A} ΔpilU	E7946 SmR, P_{tac}-tfoX, ΔluxO::miniFRT, ΔVC1807::CmR, pilA S56C, pilT ^{E204A}, ΔpilU::TmR, ΔlacZ::P_{tac}-PilT SpecR	Supp Fig 1	JLC542 (SAD2493)
P_{tac}-PilU pilT^{E204A} ΔpilU	E7946 SmR, P_{tac}-tfoX, ΔluxO::miniFRT, ΔVC1807::CmR, pilA S56C, pilT ^{E204A}, ΔpilU::TmR, , ΔlacZ::P_{tac}-PilU SpecR	Supp Fig 1	JLC543 (SAD2494)
PilU^{L199C}	E7946 SmR, ΔlacZ::lacIq, P_{tac}-tfoX, ΔluxO::miniFRT, ΔVC1807::CmR, pilA S56C, pilU ^{L199C}	Fig 2	JLC409 (SAD2495)
ΔpilT PilU^{L199C}	E7946 SmR, ΔlacZ::lacIq, P_{tac}-tfoX, ΔluxO::miniFRT, ΔVC1807::ZeoR, pilA S56C, ΔpilT::TmR, pilU ^{L199C}	Fig 2	JLC411 (SAD2496)

pilT^{K136A} pilU^{L199C}	E7946 SmR, ΔlacZ::lacIq, P_{tac}-tfoX, ΔluxO::miniFRT, ΔVC1807::CmR, pilA S56C, pilU ^{L199C}, pilT ^{K136A}	Fig 2	JLC414 (SAD2497)
pilT^{L201C}	E7946 SmR, ΔlacZ::lacIq, P_{tac}-tfoX, ΔluxO::SpecR, ΔVC1807::CmR, pilA S56C, comEA-mCherry, pilT ^{L201C} *	Fig 2	TND0989 (SAD2498)
pilT^{L201C} ΔpilU	E7946 SmR, ΔlacZ::lacIq, P_{tac}-tfoX, ΔluxO::SpecR, ΔVC1807::CmR, pilA S56C, comEA-mCherry, pilT ^{L201C}, ΔpilU::TmR *	Fig 2	TND1140 (SAD2499)
pilU^{K134A}	E7946 SmR, ΔlacZ::lacIq, P_{tac}-tfoX, ΔluxO::miniFRT, ΔVC1807::KanR, pilA S56C, pilU ^{K134A}	Fig 2	TND1092 (SAD2500)
pilT^{K136A} pilU^{K134A}	E7946 SmR, ΔlacZ::lacIq, P_{tac}-tfoX, ΔluxO::miniFRT, ΔVC1807::KanR, pilA S56C, pilT ^{K136A}, pilU ^{K134A}	Fig 2	TND1094 (SAD2501)
^{6X}His-pilU, pilT^{K136A}	E7946 SmR, ΔlacZ::lacIq, P_{tac}-tfoX, ΔluxO::miniFRT, ΔVC1807::CmR, pilA S56C, ^{6X}His-pilU, pilT ^{K136A}	Supp Fig 3	JLC298 (SAD2502)
^{6X}His-pilT	E7946 SmR, ΔlacZ::lacIq, P_{tac}-tfoX, ΔluxO::miniFRT, ΔVC1807::CmR, pilA S56C, ^{6X}His-pilT	Supp Fig 3	HKQ046 (SAD2503)
^{6X}His-pilU	E7946 SmR, ΔlacZ::lacIq, P_{tac}-tfoX, ΔluxO::miniFRT, ΔVC1807::CmR, pilA S56C, ^{6X}His-pilU	Supp Fig 3	JLC398 (SAD2504)
pilT^{K136A} 3xFLAG-pilU	E7946 SmR, ΔlacZ::lacIq, P_{tac}-tfoX, ΔluxO::miniFRT, ΔVC1807::CmR, pilA S56C, pilT ^{K136A}, 3xFLAG-pilU	Supp Fig 3	JLC396 (SAD2505)
3xFLAG-pilU	E7946 SmR, ΔlacZ::lacIq, P_{tac}-tfoX, ΔluxO::miniFRT, ΔVC1807::CmR, pilA S56C, 3xFLAG-pilU	Supp Fig 3	JLC326 (SAD2506)
3xFLAG-pilU^{K134A}	E7946 SmR, ΔlacZ::lacIq, P_{tac}-tfoX, ΔluxO::miniFRT, ΔVC1807::CmR, pilA S56C, 3xFLAG-pilU^{K134A}	Supp Fig 3	JLC316 (SAD2507)
3xFLAG-pilT	E7946 SmR, ΔlacZ::lacIq, P_{tac}-tfoX, ΔluxO::miniFRT, ΔVC1807::CmR, pilA S56C, 3xFLAG-pilT	Supp Fig 3	JLC310 (SAD2508)
3xFLAG-pilT^{K136A}	E7946 SmR, ΔlacZ::lacIq, P_{tac}-tfoX, ΔluxO::miniFRT, ΔVC1807::CmR, pilA S56C, 3xFLAG-pilT ^{K136A}	Supp Fig 3	JLC349 (SAD2509)
^{6X}His-pilU (purification)	BL21 DE3 pHisTev-6xHis-pilU	Fig 4	HKQ051 (SAD2510)
^{6X}His-pilU^{K134A} (purification)	BL21 DE3 pHisTev-6xHis-pilU ^{K134A}	Supp Fig 3	SAD2316
^{6X}His-pilT (purification)	BL21 DE3 pHisTev-6xHis-pilT	Fig 4	TND1277 (SAD2511)
^{6X}His-pilT^{K136A} (purification)	BL21 DE3 pHisTev-6xHis-pilT ^{K136A}	Supp Fig 3	TND1281 (SAD2512)
PilT x PilT	BTH101 pUT18C-PilT (CarbR), pKT25-PilT (KanR)	Fig 3, Supp Fig 2	JLC465

PilU x PilT	BTH101 pUT18C-PilU (CarbR), pKT25-PilT (KanR)	Fig 3, Supp Fig 2	JLC466
PilT^{K136A} x PilT	BTH101 pUT18C-PilT ^{K136A} (CarbR), pKT25-PilT (KanR)	Fig 3, Supp Fig 2	JLC467
PilU^{K134A} x PilT	BTH101 pUT18C-PilU ^{K134A} (CarbR), pKT25-PilT (KanR)	Fig 3, Supp Fig 2	JLC468
PilC x PilT	BTH101 pUT18C-PilC (CarbR), pKT25-PilT (KanR)	Fig 3, Supp Fig 2	JLC469
vector x PilT	BTH101 pUT18C-vector (CarbR), pKT25-PilT (KanR)	Fig 3, Supp Fig 2	JLC470
PilT x PilU	BTH101 pUT18C-PilT (CarbR), pKT25-PilU (KanR)	Fig 3, Supp Fig 2	JLC471
PilU x PilU	BTH101 pUT18C-PilU (CarbR), pKT25-PilU (KanR)	Fig 3, Supp Fig 2	JLC472
PilT^{K136A} x PilU	BTH101 pUT18C-PilT ^{K136A} (CarbR), pKT25-PilU (KanR)	Fig 3, Supp Fig 2	JLC473
PilU^{K134A} x PilU	BTH101 pUT18C-PilU ^{K134A} (CarbR), pKT25-PilU (KanR)	Fig 3, Supp Fig 2	JLC474
PilC x PilU	BTH101 pUT18C-PilC (CarbR), pKT25-PilU (KanR)	Fig 3, Supp Fig 2	JLC475
vector x PilU	BTH101 pUT18C-vector (CarbR), pKT25-PilU (KanR)	Fig 3, Supp Fig 2	JLC476
PilT x PilT^{K136A}	BTH101 pUT18C-PilT (CarbR), pKT25-PilT ^{K136A} (KanR)	Fig 3, Supp Fig 2	JLC477
PilU x PilT^{K136A}	BTH101 pUT18C-PilU (CarbR), pKT25-PilT ^{K136A} (KanR)	Fig 3, Supp Fig 2	JLC478
PilT^{K136A} x PilT^{K136A}	BTH101 pUT18C-PilT ^{K136A} (CarbR), pKT25-PilT ^{K136A} (KanR)	Fig 3, Supp Fig 2	JLC479
PilU^{K134A} x PilT^{K136A}	BTH101 pUT18C-PilU ^{K134A} (CarbR), pKT25-PilT ^{K136A} (KanR)	Fig 3, Supp Fig 2	JLC480
PilC x PilT^{K136A}	BTH101 pUT18C-PilC (CarbR), pKT25-PilT ^{K136A} (KanR)	Fig 3, Supp Fig 2	JLC481

vector x PilT^{K136A}	BTH101 pUT18C-vector (CarbR), pKT25-PilT ^{K136A} (KanR)	Fig 3, Supp Fig 2	JLC482
PilT x PilU^{K134A}	BTH101 pUT18C-PilT (CarbR), pKT25-PilU ^{K134A} (KanR)	Fig 3, Supp Fig 2	JLC483
PilU x PilU^{K134A}	BTH101 pUT18C-PilU (CarbR), pKT25-PilU ^{K134A} (KanR)	Fig 3, Supp Fig 2	JLC484
PilT^{K136A} x PilU^{K134A}	BTH101 pUT18C-PilT ^{K136A} (CarbR), pKT25-PilU ^{K134A} (KanR)	Fig 3, Supp Fig 2	JLC485
PilU^{K134A} x PilU^{K134A}	BTH101 pUT18C-PilU ^{K134A} (CarbR), pKT25-PilU ^{K134A} (KanR)	Fig 3, Supp Fig 2	JLC486
PilC x PilU^{K134A}	BTH101 pUT18C-PilC (CarbR), pKT25-PilU ^{K134A} (KanR)	Fig 3, Supp Fig 2	JLC487
vector x PilU^{K134A}	BTH101 pUT18C-vector (CarbR), pKT25-PilU ^{K134A} (KanR)	Fig 3, Supp Fig 2	JLC488
PilT x PilC	BTH101 pUT18C-PilT (CarbR), pKT25-PilC (KanR)	Fig 3, Supp Fig 2	JLC489
PilU x PilC	BTH101 pUT18C-PilU (CarbR), pKT25-PilC (KanR)	Fig 3, Supp Fig 2	JLC490
PilT^{K136A} x PilC	BTH101 pUT18C-PilT ^{K136A} (CarbR), pKT25-PilC (KanR)	Fig 3, Supp Fig 2	JLC491
PilU^{K134A} x PilC	BTH101 pUT18C-PilU ^{K134A} (CarbR), pKT25-PilC (KanR)	Fig 3, Supp Fig 2	JLC492
PilC x PilC	BTH101 pUT18C-PilC (CarbR), pKT25-PilC (KanR)	Fig 3, Supp Fig 2	JLC493
vector x PilC	BTH101 pUT18C-vector (CarbR), pKT25-PilC (KanR)	Fig 3, Supp Fig 2	JLC494
PilT x vector	BTH101 pUT18C-PilT (CarbR), pKT25-vector (KanR)	Fig 3, Supp Fig 2	JLC495
PilU x vector	BTH101 pUT18C-PilU (CarbR), pKT25-vector (KanR)	Fig 3, Supp Fig 2	JLC496
PilT^{K136A} x vector	BTH101 pUT18C-PilT ^{K136A} (CarbR), pKT25-vector (KanR)	Fig 3, Supp Fig 2	JLC497

PilU^{K134A} x vector	BTH101 pUT18C-PilU ^{K134A} (CarbR), pKT25-vector (KanR)	Fig 3, Supp Fig 2	JLC498
PilC x vector	BTH101 pUT18C-PilC (CarbR), pKT25-vector (KanR)	Fig 3, Supp Fig 2	JLC499
vector x vector	BTH101 pUT18C-vector (CarbR), pKT25-vector (KanR)	Fig 3, Supp Fig 2	JLC500
Zip x Zip	BTH101 pUT18C-Leucine Zipper (CarbR), pKT25-Leucine Zipper (KanR)	Fig 3, Supp Fig 2	JLC501
Parent (P_{const}-tfoX)	E7946 SmR, ΔlacZ::lacIq, P_{const}-tfoX, ΔluxO::miniFRT, ΔVC1807::KanR, pilA S56C	Fig 4, Supp Fig 3	TND1076 (SAD2513)
ΔpilU	E7946 SmR, ΔlacZ::lacIq, P_{const}-tfoX, ΔluxO::miniFRT, ΔVC1807::KanR, pilA S56C, ΔpilU::TmR	Fig 4, Supp Fig 3	JLC251 (SAD2514)
ΔpilT	E7946 SmR, ΔlacZ::lacIq, P_{const}-tfoX, ΔluxO::miniFRT, ΔVC1807::KanR, pilA S56C, ΔpilT::TmR	Fig 4, Supp Fig 3	JLC250 (SAD2515)
pilT^{K136A}	E7946 SmR, ΔlacZ::lacIq, P_{const}-tfoX, ΔluxO::miniFRT, ΔVC1807::CmR, pilA S56C, pilT ^{K136A}	Fig 4	JLC299 (SAD2516)
P_{tac}-pilT^{K136A}	E7946 SmR, P_{const}-tfoX, ΔluxO::miniFRT, ΔVC1807::KanR, pilA S56C, ΔlacZ::P_{tac} -pilT ^{K136A} SpecR	Fig 4	JLC277 (SAD2517)
ΔpilU P_{tac}-pilT^{K136A}	E7946 SmR, P_{const}-tfoX, ΔluxO::miniFRT, ΔVC1807::KanR, pilA S56C, ΔpilU::TmR, ΔlacZ::Ptac-pilT ^{K136A} SpecR	Fig 4	SAD2243
P_{tac}-pilU^{K134A}	E7946 SmR, P_{const}-tfoX, ΔluxO::miniFRT, ΔVC1807::KanR, pilA S56C, ΔlacZ::P_{tac}-pilU ^{K134A} SpecR	Fig 4	JLC282 (SAD2518)
pilT^{K136A} P_{tac}-pilU^{K134A}	E7946 SmR, P_{const}-tfoX, ΔluxO::miniFRT, ΔVC1807::CmR, pilA S56C, ΔlacZ::P_{tac}-pilU ^{K134A} SpecR, pilT ^{K136A}	Fig 4	JLC300 (SAD2519)
P_{tac}-pilT^{K136A} P_{tac}-pilU^{K134A}	E7946 SmR, P_{const}-tfoX, ΔluxO::miniFRT, pilA S56C, ΔlacZ::P_{tac}-pilU ^{K134A} SpecR, ΔVC1807::Ptac-pilT ^{K136A} CmR	Fig 4	SAD2242
P_{tac}-3xFLAG-PilU	E7946 SmR, P_{const}-tfoX, ΔluxO::miniFRT, ΔVC1807::KanR, pilA S56C, ΔlacZ::P_{tac}-3xFLAG-pilU SpecR	Supp Fig 3	JLC333 (SAD2520)
P_{tac}-3x FLAG-PilU ^{K134A}	E7946 SmR, P_{const}-tfoX, ΔluxO::miniFRT, ΔVC1807::KanR, pilA S56C, ΔlacZ::P_{tac}-3xFLAG-pilU ^{K134A} SpecR	Supp Fig 3	JLC336 (SAD2521)
P_{tac}-3x FLAG-PilT	E7946 SmR, ΔlacZ::lacIq, P_{const}-tfoX, ΔluxO::miniFRT, ΔVC1807::KanR, pilA S56C, ΔlacZ::P_{tac}-3xFLAG-pilT SpecR	Supp Fig 3	JLC337 (SAD2522)

P_{tac}-3x FLAG-PilT^{K136A}	E7946 SmR, P_{const}-tfoX, ΔluxO::miniFRT, ΔVC1807::KanR, pilA S56C, ΔlacZ::P_{tac}-3xFLAG-pilT ^{K136A} SpecR	Supp Fig 3	JLC353 (SAD2523)
ΔpilA	E7946 SmR, ΔlacZ::lacIq, P_{const}-tfoX, ΔluxO::miniFRT, ΔVC1807::TmR, ΔpilA::miniFRT	Supp Fig 3	TND1357 (SAD2524)
ΔpilTU	E7946 SmR, ΔlacZ::lacIq, P_{const}-tfoX, ΔluxO::miniFRT, ΔVC1807::KanR, pilA S56C, ΔpilTU::TmR	Supp Fig 3	JLC252 (SAD2525)
pilU^{K134A}	E7946 SmR, ΔlacZ::lacIq, P_{const}-tfoX, ΔluxO::miniFRT, ΔVC1807::CmR, pilA S56C, pilU ^{K134A}	Supp Fig 3	JLC308 (SAD2526)
pilT^{K136A} ΔpilU	E7946 SmR, ΔlacZ::lacIq, P_{const}-tfoX, ΔluxO::miniFRT, ΔVC1807::KanR, pilA S56C, pilT ^{K136A}, ΔpilU::TmR	Supp Fig 3	JLC447 (SAD2527)
pilT^{K136A} pilU^{K134A}	E7946 SmR, ΔlacZ::lacIq, P_{const}-tfoX, ΔluxO::miniFRT, ΔVC1807::KanR, pilA S56C, pilT ^{K136A}, pilU ^{K134A}	Supp Fig 3	JLC307 (SAD2528)
P_{tac}-pilT	E7946 SmR, P_{const}-tfoX, ΔluxO::miniFRT, ΔVC1807::KanR, pilA S56C, ΔlacZ::P_{tac}-pilT SpecR	Supp Fig 3	JLC279 (SAD2529)
ΔpilT P_{tac}-pilT	E7946 SmR, P_{const}-tfoX, ΔluxO::miniFRT, ΔVC1807::KanR, pilA S56C, ΔlacZ::P_{tac}-pilT SpecR, ΔpilT::TmR	Supp Fig 3	JLC280 (SAD2530)
P_{tac}-pilU	E7946 SmR, P_{const}-tfoX, ΔluxO::miniFRT, ΔVC1807::KanR, pilA S56C, ΔlacZ::P_{tac}-pilU SpecR	Supp Fig 3	JLC283 (SAD2531)
pilT^{K136A} ΔpilU P_{tac}-pilU	E7946 SmR, P_{const}-tfoX, ΔluxO::miniFRT, ΔVC1807::KanR, pilA S56C, ΔlacZ::P_{tac}-pilU SpecR, pilT ^{K136A}, ΔpilU::TmR	Supp Fig 3	JLC449 (SAD2532)
ΔpilT P_{tac}-3xFLAG-pilT	E7946 SmR, P_{const}-tfoX, ΔluxO::miniFRT, ΔVC1807::KanR, pilA S56C, ΔlacZ::P_{tac}-3x-FLAG-pilT SpecR, ΔpilT::TmR	Supp Fig 3	JLC373 (SAD2533)
pilT^{K136A} ΔpilU P_{tac}-3x FLAG-pilU	E7946 SmR, P_{const}-tfoX, ΔluxO::miniFRT, ΔVC1807::KanR, pilA S56C, ΔlacZ::P_{tac}-3x-FLAG-pilU SpecR, pilT ^{K136A}, ΔpilU::TmR	Supp Fig 3	JLC448 (SAD2534)
ΔpilU P_{tac}-3x FLAG-PilT^{K136A}	E7946 SmR, P_{const}-tfoX, ΔluxO::miniFRT, ΔVC1807::KanR, pilA S56C, ΔlacZ::P_{tac}-3xFLAG-pilT ^{K136A} SpecR, ΔpilU::TmR	Supp Fig 3	JLC379 (SAD2535)
pilT^{K136A} P_{tac}-3x FLAG-pilU^{K134A}	E7946 SmR, P_{const}-tfoX, ΔluxO::miniFRT, ΔVC1807::KanR, pilA S56C, ΔlacZ::P_{tac}-3x-FLAG-pilU ^{K134A} SpecR, pilT ^{K136A}	Supp Fig 3	JLC380 (SAD2536)
Parent (*A. baylyi*)	ADP1, comP T129C	Fig 5	TND0428 (SAD2537)
PilT^{K136A} ΔpilU	ADP1, comP T129C, PilT ^{K136A} ΔpilU::ZeoR	Fig 5	TND1135 (SAD2538)
PilT^{K136A} PilU^{K137A}	ADP1, , comP T129C, PilT ^{K136A} PilU ^{K137A}	Fig 5	TND1142 (SAD2539)
PilT^{K136A}	ADP1, comP T129C, PilT ^{K136A}	Fig 5	TND1122 (SAD2540)

$PilU^{K137A}$	ADP1, comP T129C, PilU ^{K137A}	Fig 5	TND1111 (SAD2541)
ΔpilT	ADP1, comP T129C, ΔpilT::SpecR	Fig 5	TND0779 (SAD2542)
ΔpilU	ADP1, comP T129C, ΔpilU::ZeoR	Fig 5	TND0798 (SAD2543)
ΔpilTU	ADP1, comP T129C, ΔpilTU::ZeoR	Fig 5	TND0799 (SAD2544)
ΔcomP	ADP1, ΔcomP::SpecR	Fig 5	TND0115 (SAD2545)
P_{tac}-pilT	ADP1, comP T129C, pMMB67EH- P_{tac}-pilT	Supp Fig 4	TND1703 (SAD2546)
P_{tac}-pilT ΔpilTU	ADP1, comP T129C, pMMB67EH- P_{tac}-pilT, ΔpilTU::ZeoR	Supp Fig 4	TND1704 (SAD2547)
P_{tac}-pilT ΔpilT	ADP1, comP T129C, pMMB67EH- P_{tac}-pilT, ΔpilT::SpecR	Supp Fig 4	TND1705 (SAD2548)
P_{tac}-pilT ΔpilU	ADP1, comP T129C, pMMB67EH- P_{tac}-pilT, ΔpilU::ZeoR	Supp Fig 4	TND1706 (SAD2549)
P_{tac}-pilT $pilT^{K136A}$	ADP1, comP T129C, pMMB67EH- P_{tac}-pilT, pilT ^{K136A}	Supp Fig 4	TND1707 (SAD2550)
P_{tac}-pilT $pilT^{K136A}$ ΔpilU	ADP1, comP T129C, pMMB67EH- P_{tac}-pilT, pilT ^{K136A}, ΔpilU::ZeoR	Supp Fig 4	TND1708 (SAD2551)
P_{tac}-pilT $pilU^{K137A}$	ADP1, comP T129C, pMMB67EH- P_{tac}-pilT, pilU ^{K137A}	Supp Fig 4	TND1709 (SAD2552)
P_{tac}-pilT $pilT^{K136A}$ $pilU^{K137A}$	ADP1, comP T129C, pMMB67EH- P_{tac}-pilT, pilT ^{K136A}, pilU ^{K137A}	Supp Fig 4	TND1710 (SAD2553)
P_{tac}-pilU	ADP1, comP T129C, pMMB67EH- P_{tac}-pilU	Supp Fig 4	TND1711 (SAD2554)
P_{tac}-pilU ΔpilTU	ADP1, comP T129C, pMMB67EH- P_{tac}-pilU, ΔpilTU::ZeoR	Supp Fig 4	TND1712 (SAD2555)
P_{tac}-pilU ΔpilT	ADP1, comP T129C, pMMB67EH- P_{tac}-pilU, ΔpilT::SpecR	Supp Fig 4	TND1713 (SAD2556)
P_{tac}-pilU ΔpilU	ADP1, comP T129C, pMMB67EH- P_{tac}-pilU, ΔpilU::ZeoR	Supp Fig 4	TND1714 (SAD2557)
P_{tac}-pilU $pilT^{K136A}$	ADP1, comP T129C, pMMB67EH- P_{tac}-pilU, pilT ^{K136A}	Supp Fig 4	TND1715 (SAD2558)
P_{tac}-pilU $pilT^{K136A}$ ΔpilU	ADP1, comP T129C, pMMB67EH- P_{tac}-pilU, pilT ^{K136A}, ΔpilU::ZeoR	Supp Fig 4	TND1716 (SAD2559)
P_{tac}-pilU $pilU^{K137A}$	ADP1, comP T129C, pMMB67EH- P_{tac}-pilU, pilU ^{K137A}	Supp Fig 4	TND1717 (SAD2560)
P_{tac}-pilU $pilT^{K136A}$ $pilU^{K137A}$	ADP1, comP T129C, pMMB67EH- P_{tac}-pilU, pilT ^{K136A}, pilU ^{K137A}	Supp Fig 4	TND1718 (SAD2561)
Parent	E7946 SmR, ΔlacZ::lacIq, P_{tac}-tfoX, ΔluxO::miniFRT, ΔVC1807::CmR, pilA S56C, comEA-mCherry	Supp Fig 5	TND0904 (SAD2636)
ΔMSHA ΔTCP	E7946 SmR, ΔlacZ::lacIq, P_{tac}-tfoX, ΔluxO::miniFRT, ΔVC1807::CmR, pilA S56C, comEA-mCherry, ΔMSHA::CarbR, ΔTCP::ZeoR	Supp Fig 5	SAD2087 (SAD2637)

ΔpilTU	E7946 SmR, ΔlacZ::lacIq, P_{tac}-tfoX, ΔluxO::miniFRT, ΔVC1807::CmR, pilA S56C, comEA-mCherry, ΔpilTU::TmR	Supp Fig 5	JLC765 (SAD2638)
ΔpilTU ΔMSHA ΔTCP	E7946 SmR, ΔlacZ::lacIq, P_{tac}-tfoX, ΔluxO::miniFRT, ΔVC1807::CmR, pilA S56C, comEA-mCherry, ΔpilTU::TmR, ΔMSHA::CarbR, ΔTCP::ZeoR	Supp Fig 5	JLC832 (SAD2639)

* comEA-mCherry does not affect natural transformation or retraction rates [17].
