# Supplementary material for: PilT and PilU are homohexameric ATPases that coordinate to retract type IVa pili
Source: PLoS Genet. 2019 Oct 18;15(10):e1008448. doi: 10.1371/journal.pgen.1008448 (PMC6821130; doi:10.1371/journal.pgen.1008448)
Supplement: S2 Table — (PDF) [file pgen.1008448.s008.pdf]

S2 Table. Primers used in this study

Primer name	Sequence (5' –> 3')	Description
BBC374	TGGCAAAAAGCGAGAGAAGAAG	ΔluxO F1
BBC375	gtcgacggatccccggaatCATGAGGACATATTTTGTTTT CTGC	ΔluxO R1
BBC376	gaagcagctccagcctacaTAAGCGATGAGAGAATGGAT CAAC	ΔluxO F2
BBC377	TCACACCCGAATTTCCATCATGC	ΔluxO R2
ABD344	GATTAGCAACGATTCTAGCGCAGGAG	ΔVC1807 F1
ABD340	gtcgacggatccccggaatACGTTTCATTAGTCACCTCTA TTGT TAACTTGTTC	ΔVC1807 R1
ABD341	gaagcagctccagcctacaTAGTCGAAAATAAAAAAAAG AGG CTCGCCTC	ΔVC1807 F2
ABD345	CTTGCTAACCGTTGGTGTTACCAGC	ΔVC1807 R2
pilA S81C F	AAAACTAATATTGAAGATTATATTGCGACAGAAG GC TgTTTTCCTGCAACAACTGCAGG	pilA S56C F
pilA R2	CATTAATCGCGGTTTCAAAGTGCA	pilA S56C R2
DOG0400	ACTTCTGGCTGAAGGTCAATTTTC	ΔpilT/U F1
DOG0401	gtcgacggatccccggaatCATTTAAATTCCTTAATAAAG TCTG GC	ΔpilT R1
DOG0402	gaagcagctccagcctacaTAGGTAGGTAAAGACAGATG GAG	ΔpilT F2
DOG0403	TCACGTGTTCGGCCAAAATC	ΔpilT/U R2
HQP0001	GGGCTCGGGTgctTCGACGACCTTGGCG	PilT ^{K136A} R1
HQP0002	CGCCAAGGTCGTCGAagcACCCGAGCCC	PilT ^{K136A} F2
BBC2508	GTTTCTTGGTCACGCAGagCACCGACGAGGATCA C	PilT ^{E204A} R1
BBC2509	GTGATCCTCGTCGGTGctCTGCGTGACCAAGAAA C	PilT ^{E204A} F2
BBC401	ACCAGCAAAGCTAATAAAATCGAG	ΔpilA F1
BBC402	gtcgacggatccccggaatGAGCATATGCCTTGCTACAC AAG	ΔpilA R1
BBC403	gaagcagctccagcctacaACTGCAGGTGCAACAATTAA CTAA	ΔpilA F2
BBC404	CGCCATACTAACCCAATACACTC	ΔpilA R2
BBC1752	CTGAATGATTTCCATGAGACG	ΔflaA F1
BBC1753	gtcgacggatccccggaatCATAGTTTGCTCTCCTATCG AG	ΔflaA R1
BBC1754	gaagcagctccagcctacaTTGCAGTAGTTCACGGTACC TTC	ΔflaA F2
BBC1755	TTATACGCTCTTTTGCGTGATGG	ΔflaA R2
BBC1450	CACTCAACGAGCTCAATACG	ΔmshA F1
ABD653	gtcgacggatccccggaatCATCTCTCTTTCATGTGAATA CGCA GC	ΔmshA R1
ABD654	gaagcagctccagcctacaGCGCAATAATTTAAATATGG CTCG TGC	ΔmshA F2

BBC1453	ATAGCCTTGCTGTTCATTTTGG	ΔmshA R2
BBC2022	TGCCGATCTCGTTTATGGACG	Δvps-rbmA locus F1
BBC2023	gtcgacggatccccggaatTGCCATTTTGATTGCCTCTGG	Δvps-rbmA locus R1
BBC2024	gaagcagctccagcctacaCAAAGAGAGCCTTATTAGGCTCTC	Δvps-rbmA locus F2
BBC2025	TGAAAGAGGTTGCTCTAGAACTCG	Δvps-rbmA locus R2
BBC1457	TTTCAAGACTTTGGGCAATAG	ΔtcpA F1
BBC1968	gtcgacggatccccggaatCTGTTTTAATAATTGCATATTTATA TAACTCCACC	ΔtcpA R1
BBC1969	gaagcagctccagcctacaTTTGGTAACAGTTAATCTACACCAT TATCTTG	ΔtcpA F2
BBC1463	TTGAATTCGTCCATCATCTAAG	ΔtcpA R2
BBC1833	GATATCCATGTCCATGTCCCAG	comEA-mCherry F1
BBC1834	cttgctcactccaccacttccacctgcTAACAAGATCCTTGCGGCATTG	comEA-mCherry R1
BBC1837	acgagctgtacaagtaaGCTGCAATCATGTGTCTCG	comEA-mCherry F2
BBC1838	GTCTTTCTATCAGTTCGGACTTAGTC	comEA-mCherry R2
BBC1835	gcaggtggaagtggtggaGTGAGCAAGGGCGAGGAGG	mCherry (for comEAmCherry) F
BBC1836	gattgcagcTTACTTGTACAGCTCGTCC	mCherry (for comEAmCherry) R
DOG0405	gtcgacggatccccggaatCATCTGTCTTTACCTACCTAAAATGC	ΔpilU R1
DOG0406	gaagcagctccagcctacaTAGCGATGGTTACTTTGTGCG	ΔpilU F2
JCP048	CAAGCCCCCGATATGATTTgcATTGGTGAAATCCGCAGC	PilU ^{L199C} F
JCP049	GCTGCGGATTTCACCAATgcAAATCATATCGGGGGCTTG	PilU ^{L199C} R
BBC2287	CATTGCGCTCGGCATTGCGTGAAGACCCAGATGTGATCtgtGTCGGTGAGCTGCGTGAC	PilT ^{L201C} F2
BBC2288	CGCAATGCCGAGCGCAATG	PilT ^{L201C} R1
BBC2369	GGCTGCCATAGTGGTCGAggcACCCGAACCGGTTGCGC	PilU ^{K134A} R1
BBC2347	GCGCAACCGGTTCGGGTgccTCGACCACTATGGCAGCC	PilU ^{K134A} F2
BBC2313	gcaggtggaagtggtggaCATCATCATCATCATCACgcaggtggagcaggtgga	6xHis (for 6xHis-PilU/T) F
BBC2314	tccacctgctccacctgcGTGATGATGATGATGATGtccaccacttccacctgc	6xHis (for 6xHis-PilU/T) R
BBC2272	gcaggtggaagtggtggagattataaagaccatgatggtgactacaaggatcacgacattgattataaggatgacgatgacaaagcaggtggagcaggtgga	3xFLAG (for 3xFLAG-PilU/T) F
BBC2273	tccacctgctccacctgctttgtcatcgtcatccttataatcaatgtcgtgatccttgtagtcaccatcatggtctttataatctccaccacttccacctgc	3xFLAG (for 3xFLAG-PilU/T) R
JCP028	tccaccacttccacctgcCATCTGTCTTTACCTACCTAAAATGC	X-PilU (N-term tags) R1

JCP029	gcaggtggagcaggtggaGAGTTGAATCAATATCTGGA TGGC	X-PilU (N-term tags OR BACTH TXX-pilU) F2
JCP026	tccaccacttccacctgcCATTTAAATTCCTTAATAAAGTC TGGCTATG	X-PilT (N-term tags) R1
JCP027	gcaggtggagcaggtggaGATATCGCTGAGTTACTGGA ATTTAG	X-PilT (N-term tags or BACTH TXX-pilT) F2
ABD332	GGCTGAACGTGGTTGTCGAAAATGAC	P_{tac}-PilT/U @lacZ F1
BBC244	CCCGGGATCCTGTGTGAAATTGTTATCCGC	P_{tac}-PilT/U @lacZ or @VC1807 R1
ABD255	TTAAGCTACTAAAGCGTAGTTTTCG	P_{tac}-PilT/U @lacZ F2
ABD256	CAATTTCACACAGGATCCCGGGAGGAGGTAACG TAATGGTGAGCAAAGGCGAAG	P_{tac}-PilT/U @lacZ R2
BBC1723	caatttcacacaggatcccgggAGGAGGTATTTAAATGGA TATCGCTGAGTTAC	Amplify pilT (for P_{tac}-pilT) F
BBC1724	tgtaggctggagctgcttcCTAAAATGCTTTTAAATCCAAC TCG	Amplify pilT (for P_{tac}-pilT OR BACTH TXX-pilT) R
BBC1725	caatttcacacaggatcccgggAGGAGGTAGACAGATGGA GTTGAATCAATATC	Amplify pilU (for P_{tac}-pilU) F
BBC1726	tgtaggctggagctgcttcCTACTCCATATCAATCTTCACA TTC	Amplify pilU (for P_{tac}-pilU OR BACTH TXX-pilU) R
ABD342	ATTTTTCAGTTGGCCTACAATGCTTTCC	P_{tac}-PilT/U @VC1807 F1
BBC719	CACCATACCCACGCCGAAACAAGGATTTTGAATT AAACGTTTCATTAGTC	P_{tac}-PilT/U @VC1807 R1
BBC243	TTGTTTCGGCGTGGGTATGGTG	P_{tac}-PilT/U @VC1807 F2
BBC2667	tccaccacttccacctgcCATTACGTTACCTCCTCCCG	P_{tac}-X-PilT/U (X is 3xFLAG or 6xHis) R
BBC2513	gaagcagctccagcctacaGATCCCCGGGTACCTAAGT AAC	BACTH pKT25-X F
BBC2512	tccacctgctccacctgcCTCTAGAGTCGACCCTGCAG	BACTH pKT25-X R
BBC2515	gaagcagctccagcctacaCCGAGCTCGAATTCATCGAT ATAAC	BACTH pUT18C-X F
BBC2514	tccacctgctccacctgcCTCTAGAGTCGACCTGCAG	BACTH pUT18C-X R
BBC2518	gcaggtggagcaggtggaAAAGCGACCCAAACCTTACC	BACTH TXX-PilC F
BBC2524	tgtaggctggagctgcttcTTAACCCAATACACTCATTAA GTTAAAG	BACTH TXX-PilC R
BBC2358	GGTATATCTCCTTCTTAAAGTTAAACAAAATTATT TCTAGAGGGGAATTGTTATCC	pHisTev-X F
BBC2360	cggatccGGCTGCTAACAAAGCCCGAAAGGAAGCT GAGTTGGCTGCTGC	pHisTev-X R
BBC2478	TTTTGTTTAACTTTAAGAAGGAGATATACCATGgc aggtggaagtggtgga	Amplify 6xHis-pilT or 6xHis-PilU to clone into pHistev F
BBC2479	CCTTTCGGGCTTTGTTAGCAGCCggatccgCTAAAA TGCTTTTAAATCCAACTCG	Amplify 6xHis-pilT to clone into pHistev R
BBC2480	CCTTTCGGGCTTTGTTAGCAGCCggatccgCTACTC CATATCAATCTTCACATTCTG	Amplify 6xHis-pilU to clone into pHistev R
BBC706	CCACACATTATACGAGCCGATG	P_{const}-tfoX R1
BBC2226	catcggctcgtataatgtgtggaGGGGAACGTGATTAAAGG A	P_{const}-tfoX F2

ABD332	GGCTGAACGTGGTTGTCGAAAATGAC	ΔlacZ::lacIq F1
ABD331	GAACATGGGGTGTACGGCAGTGCtATTTAACGATGTGCGGGTTTTGCCAATCTTG	ΔlacZ::lacIq R1
ABD255	gaagcagctccagcctacaCCACAATAAGCCAGAGAGCCTTAAG	ΔlacZ::lacIq F2
ABD256	CCCAAATACGGCAACTTGGCG	ΔlacZ::lacIq R2
ABD841	GGCACTGCCGTACACCCCATGTTCCGGTACCCGACACCATCGAATG	Amplify lacIq F
ABD842	GGCTCTCTGGCTTATTGTGGGGGGAAACCTGTCGTGCCAGCTGC	Amplify lacIq R
DOG0175	AAACTATCCAGATAAGGGAAAGC	comP (*A. baylyi*) F1
CE87	GAAGTTGTCGTACATTTCCAacaTACAGCGCCACTAGCATATG	comP T129C (*A. baylyi*) R1
CE88	CATATGCTAGTGGCGCTGTATGTTGGAAATGTACGACAACTTC	comP T129C (*A. baylyi*) F2
DOG0178	AGGATCTGTAATGACGGGTTGAG	comP (*A. baylyi*) R2
DOG0176	gtcgacggatccccggaatCATAAAATTTCTCCACCAATGTTG	ΔcomP (*A. baylyi*) R1
DOG0177	gaagcagctccagcctacaTGATAGTAGTACTATATGGCTTTAAAAG	ΔcomP (*A. baylyi*) F2
BBC1934	GTTACAAAGTCAGGGACGTAAAG	pilT or pilU (*A. baylyi*) F1
BBC1935	gtcgacggatccccggaatATCCATATTTTCCCCGAAGATCG	ΔpilT (*A. baylyi*) R1
BBC1936	gaagcagctccagcctacaTAAGAATAACGCTACTCGATCTG	ΔpilT (*A. baylyi*) F2
BBC1939	GGTATTCAGATTGATCGTCAGTTAG	pilT or pilU (*A. baylyi*) R2
BBC1937	gtcgacggatccccggaatCATCGTATTGTTTTCCTCTCAGATC	ΔpilU (*A. baylyi*) R1
BBC1938	gaagcagctccagcctacaCGCCGTAATCTCAAATAAAAGAG	ΔpilU (*A. baylyi*) F2
BBC2403	GCTGCAAGGGTGGTTGAagcACCGGAACCAGTGG	$pilT^{K136A}$ (*A. baylyi*) R1
BBC2404	CCACTGGTTCCGGTgctTCAACCACCCTTGCAGC	$pilT^{K136A}$ (*A. baylyi*) F2
BBC2408	GGATGCCAAAGACGTGGAagcTCCTGTACCAGTTGC	$pilU^{K137A}$ (*A. baylyi*) R1
BBC2409	GCAACTGGTACAGGAgctTCCACGTCTTTGGCATCC	$pilU^{K137A}$ (*A. baylyi*) F2
BBC2807	caatttcacacaggatcccgggAGGAGGTGAAAATATGGATATAACAGAATTACTCG	pilT amplify for insertion into P_{tac} construct (*A. baylyi*) F
BBC2808	tgtaggctggagctgcttcTTAGAACGACTCTGGAGATTTTG	pilT amplify for insertion into P_{tac} construct or pMMB67EH (*A. baylyi*) R
BBC2809	caatttcacacaggatcccgggAGGAGGTAATACGATGGACTTTAATGATTTACTC	pilU amplify for insertion into P_{tac} construct (*A. baylyi*) F
BBC2810	tgtaggctggagctgcttcTTATTTGAGATTACGGCGTTGAC	pilU amplify for insertion into P_{tac} construct or pMMB67EH (*A. baylyi*) R

BBC2822	aatttcacacaggaaacagaattcgagctcTTGTTTCGGCGTGGGTATGGTG	Clone P_{tac}-pilT or P_{tac}-PilU into pMMB67EH (*A. baylyi*) F
BBC2824	gagctcgaattctgtttcctgtgtgaaattTTTGCCAGAACCGTTATGATGTC	Amplify pMMB67EH to clone P_{tac}-pilT or P_{tac}-pilU (*A. baylyi*) F
BBC2357	GGTACCCGGGGATCCTCTAGAGTCGACCTGCAGGCATGCAAGCTTGGC	Amplify pMMB67EH to clone P_{tac}-pilT or P_{tac}-pilU (*A. baylyi*) R
DOG0436	AAGCGTTCTGGTTCAATCACC	ΔMSHA locus (VC0399-VC0414) F1
DOG0434	gtcgacggatccccggaatCATTCTCTACCACTGCTATTTGGTTC	ΔMSHA locus (VC0399-VC0414) R1
BBC2129	gaagcagctccagcctacaTAATGATTAAGCACTCAATGGATCCAG	ΔMSHA locus (VC0399-VC0414) F2
BBC2130	TTTTGCATCAGCAAAATCACGC	ΔMSHA locus (VC0399-VC0414) R2
BBC2123	TGATGGTAACTACTATAGGGTCG	ΔTCP locus (VC0825-VC0838) F1
BBC2124	gtcgacggatccccggaatTAAATTAGGCTAGTGCCAGTCAG	ΔTCP locus (VC0825-VC0838) R1
BBC2125	gaagcagctccagcctacaACAGGAGTTGCAGAAAAATAATGG	ΔTCP locus (VC0825-VC0838) F2
BBC2126	ACTAAGATAATTGCTTCACGCATG	ΔTCP locus (VC0825-VC0838) R2
