# Supplementary material for: PilT and PilU are homohexameric ATPases that coordinate to retract type IVa pili
Source: PLoS Genet. 2019 Oct 18;15(10):e1008448. doi: 10.1371/journal.pgen.1008448 (PMC6821130; doi:10.1371/journal.pgen.1008448)
Supplement: S3 Table — (PDF) [file pgen.1008448.s009.pdf]

S3 Table. Mean values for each data set

Figure	Strain Name	Mean	Figure	Strain Name	Mean
Fig 1A	Parent	1.08E-01	S2 Fig	PilT x PilT	1.67E+02
Fig 1A	ΔpilA	1.22E-08	S2 Fig	PilU x PilT	6.10E+01
Fig 1A	ΔpilU	5.78E-02	S2 Fig	PilT^{K136A} x PilT	1.57E+02
Fig 1A	ΔpilT	3.29E-06	S2 Fig	PilU^{K134A} x PilT	8.23E+01
Fig 1A	ΔpilTΔpilU	3.91E-06	S2 Fig	PilC x PilT	1.15E+02
Fig 1A	pilT^{K136A}	6.22E-02	S2 Fig	vector x PilT	1.13E+00
Fig 1A	pilT^{K136A} ΔpilU	3.89E-06	S2 Fig	PilT x PilU	1.10E+02
Fig 1A	pilT^{E204A}	2.55E-02	S2 Fig	PilU x PilU	1.81E+02
Fig 1A	pilT$^{K136A/E204A}$	3.53E-02	S2 Fig	PilT^{K136A} x PilU	7.37E+01
Fig 1A	pilT^{E204A} ΔpilU	9.36E-06	S2 Fig	PilU^{K134A} x PilU	2.11E+02
Fig 1A	pilT$^{K136A/E204A}$ ΔpilU	4.66E-06	S2 Fig	PilC x PilU	9.71E+01
Fig 1B	Parent	2.12E-01	S2 Fig	vector x PilU	1.04E+00
Fig 1B	ΔpilU	1.57E-01	S2 Fig	PilT x PilT^{K136A}	1.56E+02
Fig 1B	ΔpilT	3.07E-03	S2 Fig	PilU x PilT^{K136A}	7.99E+00
Fig 1B	ΔpilTΔpilU	3.85E-03	S2 Fig	PilT^{K136A} x PilT^{K136A}	8.06E+01
Fig 1B	pilT^{K136A}	4.26E-02	S2 Fig	PilU^{K134A} x PilT^{K136A}	1.04E+01
Fig 1B	pilT^{K136A} ΔpilU	2.06E-03	S2 Fig	PilC x PilT^{K136A}	1.08E+02
Fig 1B	pilT^{E204A}	4.82E-02	S2 Fig	vector x PilT^{K136A}	7.32E-01
Fig 1B	pilT$^{E204A/K136A}$	6.12E-02	S2 Fig	PilT x PilU^{K134A}	7.20E+01
Fig 1B	pilT^{E204A} ΔpilU	3.58E-03	S2 Fig	PilU x PilU^{K134A}	1.72E+02
Fig 1B	pilT$^{E204A/K136A}$ ΔpilU	4.09E-03	S2 Fig	PilT^{K136A} x PilU^{K134A}	4.69E+01
Fig 1C	Parent	1.73E+01	S2 Fig	PilU^{K134A} x PilU^{K134A}	1.34E+02
Fig 1C	ΔpilU	3.16E+01	S2 Fig	PilC x PilU^{K134A}	1.10E+02
Fig 1C	ΔpilT	2.93E-02	S2 Fig	vector x PilU^{K134A}	7.14E-01
Fig 1C	ΔpilTΔpilU	1.33E-01	S2 Fig	PilT x PilC	8.69E+01
Fig 1C	pilT^{K136A}	3.43E+01	S2 Fig	PilU x PilC	5.20E+01
Fig 1C	pilT^{K136A} ΔpilU	8.75E-02	S2 Fig	PilT^{K136A} x PilC	7.43E+01
Fig 1C	pilT^{E204A}	1.41E-01	S2 Fig	PilU^{K134A} x PilC	7.25E+01
Fig 1C	pilT$^{E204A/K136A}$	1.77E+01	S2 Fig	PilC x PilC	2.55E+02
Fig 1C	pilT^{E204A}ΔpilU	5.65E-02	S2 Fig	vector x PilC	8.15E-01
Fig 1C	pilT$^{E204A/K136A}$ΔpilU	3.86E-02	S2 Fig	PilT x vector	6.59E-01
Fig 1F	Parent	1.05E+01	S2 Fig	PilU x vector	7.85E-01
Fig 1F	ΔpilT	4.93E+00	S2 Fig	PilT^{K136A} x vector	6.81E-01
Fig 1F	ΔpilU	5.08E+00	S2 Fig	PilU^{K134A} x vector	7.14E-01
Fig 1F	ΔpilTΔpilU	6.08E+00	S2 Fig	PilC x vector	7.68E-01
Fig 1H	Parent	1.93E-01	S2 Fig	vector x vector	7.22E-01
Fig 1H	ΔpilT	1.37E-02	S3-A Fig	Parent	1.46E-01

Fig 1H	ΔpilU	7.23E-02
Fig 1H	ΔpilTΔpilU	8.19E-03
Fig 2A	Parent	1.37E-01
Fig 2A	pilU^{L199C}	1.51E-01
Fig 2A	pilT^{K136A}pilU^{L199C}	9.81E-02
Fig 2A	ΔpilT pilU^{L199C}	6.79E-06
Fig 2A	pilT^{L201C}	2.04E-01
Fig 2A	pilT^{L201C} ΔpilU	6.47E-02
Fig 2A	pilT^{K136A}	1.17E-01
Fig 2A	pilU^{K134A}	6.33E-02
Fig 2A	pilT^{K136A}pilU^{K134A}	6.07E-07
Fig 2A	ΔpilU	8.80E-02
Fig 2B	Parent	2.12E-01
Fig 2B	ΔpilU	1.57E-01
Fig 2B	pilT^{K136A}	4.26E-02
Fig 2B	pilU^{K134A}	1.37E-01
Fig 2B	pilT^{K136A}pilU^{K134A}	4.77E-03
Fig 2B	pilT^{K136A} pilU^{L199C}	3.26E-02
Fig 2B	pilU^{L199C}	1.96E-01
Fig 2B	pilT^{L201C}	2.11E-01
Fig 2B	pilT^{L201C}ΔpilU	1.28E-01
Fig 2B	ΔpilT pilU^{L199C}	3.91E-03
Fig 4C	Parent	2.02E-03
Fig 4C	ΔpilU	2.57E-03
Fig 4C	ΔpilT	1.57E-06
Fig 4C	pilT^{K136A}	3.08E-03
Fig 4C	Ptac-pilT^{K136A}	5.94E-03

S3-A Fig	ΔpilT	6.94E-06
S3-A Fig	pilT^{K136A}, 6xHis-pilU	1.53E-01
S3-A Fig	6xHis-pilT	1.14E-01
S3-A Fig	6xHis-pilU	1.11E-01
S3-A Fig	pilT^{K136A}, 3xFLAG-pilU	1.14E-01
S3-A Fig	3xFLAG-pilT	1.45E-01
S3-A Fig	3xFLAG-pilU	1.23E-01
S3-A Fig	pilT^{K136A}	1.20E-01
S3-B Fig	Parent	3.75E-03
S3-B Fig	ΔpilT	4.46E-07
S3-B Fig	Ptac-3xFLAG-pilT	4.60E-03
S3-B Fig	Ptac-3xFLAG-pilT ΔpilT	4.94E-03
S3-B Fig	Ptac-3xFLAG-pilU	2.78E-03
S3-B Fig	Ptac-3xFLAG-pilU, pilT^{K136A} ΔpilU	1.66E-03
S3-B Fig	Ptac-3xFLAG-pilT^{K136A}	5.66E-03
S3-B Fig	Ptac-3xFLAG-pilU^{K134A}	4.71E-03
S3-B Fig	Ptac-3xFLAG-pilT^{K136A}, ΔpilU	1.25E-06
S3-B Fig	P$_{tac}$-3xFLAG-pilU^{K134A}, pilT^{K136A}	2.00E-06
S3-B Fig	ΔpilU	2.48E-03
S3-B Fig	pilT^{K136A}	2.24E-03
S3-D Fig	Parent	1.97E-03
S3-D Fig	ΔpilT ΔpilU	2.15E-07
S3-D Fig	pilU^{K134A}	3.90E-03
S3-D Fig	pilT^{K136A} ΔpilU	3.62E-08
S3-D Fig	pilT^{K136A} pilU^{K134A}	2.12E-07
S3-D Fig	P$_{tac}$-pilT	4.18E-03
S3-D Fig	ΔpilT Ptac-pilT	4.08E-03

Fig 4C	Ptac-pilT^{K136A} ΔpilU	2.41E-06		S3-D Fig	P$_{tac}$-pilU	4.07E-03
Fig 4C	Ptac-pilU^{K134A}	5.55E-03		S3-D Fig	pilT^{K136A} ΔpilU Ptac-pilU	1.39E-03
Fig 4C	Ptac-pilU^{K134A} pilT^{K136A}	2.46E-05		S3-D Fig	ΔpilA	6.49E-09
Fig 4C	Ptac-pilT^{K136A} Ptac-pilU^{K134A}	4.82E-06		S3-D Fig	pilT^{K136A}	1.75E-03
Fig 5	pilT^{K136A} ΔpilU	8.95E-09		S4 Fig	Parent pmmB-pilT	2.23E-02
Fig 5	pilT^{K136A} pilU^{K137A}	7.00E-09		S4 Fig	ΔpilTU pmmB-pilT	2.05E-03
Fig 5	pilT^{K136A}	1.17E-03		S4 Fig	ΔpilT pmmB-pilT	1.80E-02
Fig 5	pilU^{K137A}	2.34E-03		S4 Fig	ΔpilU pmmB-pilT	3.24E-03
Fig 5	Parent	4.10E-02		S4 Fig	pilT^{K136A} pmmB-pilT	1.68E-02
Fig 5	ΔpilT	8.52E-09		S4 Fig	pilT^{K136A} ΔpilU pmmB-pilT	2.30E-03
Fig 5	ΔpilU	3.17E-03		S4 Fig	pilU^{K137A} pmmB-pilT	1.63E-03
Fig 5	ΔpilTU	8.48E-09		S4 Fig	pilT^{K136A} pilU^{K137A} pmmB-pilT	2.19E-03
Fig 5	ΔcomP	6.53E-09		S4 Fig	Parent pmmB-pilU	8.67E-02
S1 Fig	ΔpilT ΔpilU Ptac-PilT	7.05E-02		S4 Fig	ΔpilTU pmmB-pilU	2.04E-07
S1 Fig	ΔpilT ΔpilU Ptac-PilU	7.26E-06		S4 Fig	ΔpilT pmmB-pilU	1.55E-07
S1 Fig	pilT^{K136A} ΔpilU Ptac-PilT	6.53E-02		S4 Fig	ΔpilU pmmB-pilU	2.48E-02
S1 Fig	pilT^{K136A} ΔpilU Ptac-PilU	6.27E-02		S4 Fig	pilT^{K136A} pmmB-pilU	7.66E-04
S1 Fig	pilT^{K136A} pilU^{K134A} Ptac-PilT	4.21E-02		S4 Fig	pilT^{K136A} ΔpilU pmmB-pilU	8.35E-04
S1 Fig	pilT^{K136A} pilU^{K134A} Ptac-PilU	4.65E-02		S4 Fig	pilU^{K137A} pmmB-pilU	1.07E-02
S1 Fig	Parent Ptac-PilT	6.74E-02		S4 Fig	pilT^{K136A} pilU^{K137A} pmmB-pilU	9.52E-04
S1 Fig	Parent Ptac-PilU	8.85E-02		S4 Fig	Parent pmmB-pilU	2.66E-02
S1 Fig	ΔpilT Ptac-PilT	6.55E-02		S5 Fig	Parent	3.12E-01
S1 Fig	ΔpilT Ptac-PilU	3.13E-06		S5 Fig	ΔMSHA ΔTCP	3.73E-01
S1 Fig	pilT$^{K136A/E204A}$ ΔpilU Ptac-PilT	6.32E-02		S5 Fig	ΔpilTU	1.59E-05
S1 Fig	pilT$^{K136A/E204A}$ ΔpilU Ptac-PilU	4.05E-02		S5 Fig	ΔpilTU ΔMSHA ΔTCP	3.34E-05
S1 Fig	pilT^{E204A} ΔpilU Ptac-PilT	6.02E-02				
S1 Fig	pilT^{E204A} ΔpilU Ptac-PilU	7.81E-02				
