# Supplementary material for: PilT and PilU are homohexameric ATPases that coordinate to retract type IVa pili
Source: PLoS Genet. 2019 Oct 18;15(10):e1008448. doi: 10.1371/journal.pgen.1008448 (PMC6821130; doi:10.1371/journal.pgen.1008448)
Supplement: S4 Table — (PDF) [file pgen.1008448.s010.pdf]

S4 Table. Statistical Comparisons.

Comparison	P value[#]		Information
**Figure 1A - Tukey's multiple comparison test**			
Parent vs. ΔpilA	****	<0.0001	# of families = 1
Parent vs. ΔpilU	ns	0.6432	# of comparisons per family = 55
Parent vs. ΔpilT	****	<0.0001	Alpha = 0.05
Parent vs. ΔpilTΔpilU	****	<0.0001	
Parent vs. pilT^{K136A}	ns	0.804	
Parent vs. pilT^{K136A} ΔpilU	****	<0.0001	
Parent vs. pilT^{E204A}	****	<0.0001	
Parent vs. pilT$^{K136A/E204A}$	*	0.0135	
Parent vs. pilT^{E204A} ΔpilU	****	<0.0001	
Parent vs. pilT$^{K136A/E204A}$ ΔpilU	****	<0.0001	
ΔpilA vs. ΔpilU	****	<0.0001	
ΔpilA vs. ΔpilT	****	<0.0001	
ΔpilA vs. ΔpilTΔpilU	****	<0.0001	
ΔpilA vs. pilT^{K136A}	****	<0.0001	
ΔpilA vs. pilT^{K136A} ΔpilU	****	<0.0001	
ΔpilA vs. pilT^{E204A}	****	<0.0001	
ΔpilA vs. pilT$^{K136A/E204A}$	****	<0.0001	
ΔpilA vs. pilT^{E204A} ΔpilU	****	<0.0001	
ΔpilA vs. pilT$^{K136A/E204A}$ ΔpilU	****	<0.0001	
ΔpilU vs. ΔpilT	****	<0.0001	
ΔpilU vs. ΔpilTΔpilU	****	<0.0001	
ΔpilU vs. pilT^{K136A}	ns	>0.9999	
ΔpilU vs. pilT^{K136A} ΔpilU	****	<0.0001	
ΔpilU vs. pilT^{E204A}	ns	0.0523	
ΔpilU vs. pilT$^{K136A/E204A}$	ns	0.8854	
ΔpilU vs. pilT^{E204A} ΔpilU	****	<0.0001	
ΔpilU vs. pilT$^{K136A/E204A}$ ΔpilU	****	<0.0001	
ΔpilT vs. ΔpilTΔpilU	ns	>0.9999	
ΔpilT vs. pilT^{K136A}	****	<0.0001	
ΔpilT vs. pilT^{K136A} ΔpilU	ns	>0.9999	
ΔpilT vs. pilT^{E204A}	****	<0.0001	
ΔpilT vs. pilT$^{K136A/E204A}$	****	<0.0001	
ΔpilT vs. pilT^{E204A} ΔpilU	ns	0.1222	
ΔpilT vs. pilT$^{K136A/E204A}$ ΔpilU	ns	0.994	
ΔpilTΔpilU vs. pilT^{K136A}	****	<0.0001	
ΔpilTΔpilU vs. pilT^{K136A} ΔpilU	ns	>0.9999	
ΔpilTΔpilU vs. pilT^{E204A}	****	<0.0001	
ΔpilTΔpilU vs. pilT$^{K136A/E204A}$	****	<0.0001	

ΔpilTΔpilU vs. $pilT^{E204A}$ ΔpilU	ns	0.1655	
ΔpilTΔpilU vs. $pilT^{K136A/E204A}$ ΔpilU	ns	0.9983	
$pilT^{K136A}$ vs. $pilT^{K136A}$ ΔpilU	****	<0.0001	
$pilT^{K136A}$ vs. $pilT^{E204A}$	*	0.03	
$pilT^{K136A}$ vs. $pilT^{K136A/E204A}$	ns	0.7787	
$pilT^{K136A}$ vs. $pilT^{E204A}$ ΔpilU	****	<0.0001	
$pilT^{K136A}$ vs. $pilT^{K136A/E204A}$ ΔpilU	****	<0.0001	
$pilT^{K136A}$ ΔpilU vs. $pilT^{E204A}$	****	<0.0001	
$pilT^{K136A}$ ΔpilU vs. $pilT^{K136A/E204A}$	****	<0.0001	
$pilT^{K136A}$ ΔpilU vs. $pilT^{E204A}$ ΔpilU	ns	0.1909	
$pilT^{K136A}$ ΔpilU vs. $pilT^{K136A/E204A}$ ΔpilU	ns	0.9992	
$pilT^{E204A}$ vs. $pilT^{K136A/E204A}$	ns	0.7599	
$pilT^{E204A}$ vs. $pilT^{E204A}$ ΔpilU	****	<0.0001	
$pilT^{E204A}$ vs. $pilT^{K136A/E204A}$ ΔpilU	****	<0.0001	
$pilT^{K136A/E204A}$ vs. $pilT^{E204A}$ ΔpilU	****	<0.0001	
$pilT^{K136A/E204A}$ vs. $pilT^{K136A/E204A}$ ΔpilU	****	<0.0001	
$pilT^{E204A}$ ΔpilU vs. $pilT^{K136A/E204A}$ ΔpilU	ns	0.6405	
**Figure 1B - Tukey's multiple comparison test**			
Parent vs. ΔpilU	****	<0.0001	# of families = 1
Parent vs. ΔpilT	****	<0.0001	# of comparisons per family = 45
Parent vs. ΔpilTΔpilU	****	<0.0001	Alpha = 0.05
Parent vs. $pilT^{K136A}$	****	<0.0001	
Parent vs. $pilT^{K136A}$ ΔpilU	****	<0.0001	
Parent vs. $pilT^{E204A}$	****	<0.0001	
Parent vs. $pilT^{E204A/K136A}$	****	<0.0001	
Parent vs. $pilT^{E204A}$ ΔpilU	****	<0.0001	
Parent vs. $pilT^{E204A/K136A}$ ΔpilU	****	<0.0001	
ΔpilU vs. ΔpilT	****	<0.0001	
ΔpilU vs. ΔpilTΔpilU	****	<0.0001	
ΔpilU vs. $pilT^{K136A}$	****	<0.0001	
ΔpilU vs. $pilT^{K136A}$ ΔpilU	****	<0.0001	
ΔpilU vs. $pilT^{E204A}$	****	<0.0001	
ΔpilU vs. $pilT^{E204A/K136A}$	****	<0.0001	
ΔpilU vs. $pilT^{E204A}$ ΔpilU	****	<0.0001	
ΔpilU vs. $pilT^{E204A/K136A}$ ΔpilU	****	<0.0001	
ΔpilT vs. ΔpilTΔpilU	ns	>0.9999	
ΔpilT vs. $pilT^{K136A}$	ns	0.0674	
ΔpilT vs. $pilT^{K136A}$ ΔpilU	ns	>0.9999	
ΔpilT vs. $pilT^{E204A}$	ns	0.4312	
ΔpilT vs. $pilT^{E204A/K136A}$	***	0.0005	
ΔpilT vs. $pilT^{E204A}$ ΔpilU	ns	>0.9999	
ΔpilT vs. $pilT^{E204A/K136A}$ ΔpilU	ns	>0.9999	

ΔpilTΔpilU vs. pilT^{K136A}	**	0.0027	
ΔpilTΔpilU vs. pilT^{K136A} ΔpilU	ns	>0.9999	
ΔpilTΔpilU vs. pilT^{E204A}	ns	0.3118	
ΔpilTΔpilU vs. pilT^{E204A}/^{K136A}	****	<0.0001	
ΔpilTΔpilU vs. pilT^{E204A} ΔpilU	ns	>0.9999	
ΔpilTΔpilU vs. pilT^{E204A}/^{K136A} ΔpilU	ns	>0.9999	
pilT^{K136A} vs. pilT^{K136A} ΔpilU	ns	0.0538	
pilT^{K136A} vs. pilT^{E204A}	ns	>0.9999	
pilT^{K136A} vs. pilT$^{E204A/K136A}$	ns	0.1608	
pilT^{K136A} vs. pilT^{E204A} ΔpilU	**	0.0034	
pilT^{K136A} vs. pilT^{E204A}/^{K136A} ΔpilU	ns	0.2706	
pilT^{K136A} ΔpilU vs. pilT^{E204A}	ns	0.3977	
pilT^{K136A} ΔpilU vs. pilT^{E204A}/^{K136A}	***	0.0004	
pilT^{K136A} ΔpilU vs. pilT^{E204A} ΔpilU	ns	>0.9999	
pilT^{K136A} ΔpilU vs. pilT^{E204A}/^{K136A} ΔpilU	ns	>0.9999	
pilT^{E204A} vs. pilT$^{E204A/K136A}$	ns	0.9989	
pilT^{E204A} vs. pilT^{E204A} ΔpilU	ns	0.3122	
pilT^{E204A} vs. pilT^{E204A}/^{K136A} ΔpilU	ns	0.5871	
pilT^{E204A}/^{K136A} vs. pilT^{E204A} ΔpilU	****	<0.0001	
pilT^{E204A}/^{K136A} vs. pilT^{E204A}/^{K136A} ΔpilU	*	0.0109	
pilT^{E204A} ΔpilU vs. pilT^{E204A}/^{K136A} ΔpilU	ns	>0.9999	
**Figure 1C - Tukey's multiple comparison test**			
Parent vs. ΔpilU	ns	>0.9999	# of families = 1
Parent vs. ΔpilT	****	<0.0001	# of comparisons per family = 45
Parent vs. ΔpilTΔpilU	****	<0.0001	Alpha = 0.05
Parent vs. pilT^{K136A}	ns	0.9963	
Parent vs. pilT^{K136A} ΔpilU	***	0.0002	
Parent vs. pilT^{E204A}	***	0.0006	
Parent vs. pilT$^{E204A/K136A}$	ns	>0.9999	
Parent vs. pilT^{E204A} ΔpilU	****	<0.0001	
Parent vs. pilT^{E204A}/^{K136A} ΔpilU	****	<0.0001	
ΔpilU vs. ΔpilT	****	<0.0001	
ΔpilU vs. ΔpilTΔpilU	****	<0.0001	
ΔpilU vs. pilT^{K136A}	ns	>0.9999	
ΔpilU vs. pilT^{K136A} ΔpilU	****	<0.0001	
ΔpilU vs. pilT^{E204A}	***	0.0002	
ΔpilU vs. pilT^{E204A}/^{K136A}	ns	>0.9999	
ΔpilU vs. pilT^{E204A} ΔpilU	****	<0.0001	
ΔpilU vs. pilT^{E204A}/^{K136A} ΔpilU	****	<0.0001	
ΔpilT vs. ΔpilTΔpilU	ns	0.9878	
ΔpilT vs. pilT^{K136A}	****	<0.0001	
ΔpilT vs. pilT^{K136A} ΔpilU	ns	0.9085	

ΔpilT vs. pilT^{E204A}	ns	0.6143	
ΔpilT vs. pilT^{E204A}/^{K136A}	****	<0.0001	
ΔpilT vs. pilT^{E204A} ΔpilU	ns	>0.9999	
ΔpilT vs. pilT^{E204A}/^{K136A} ΔpilU	ns	>0.9999	
ΔpilTΔpilU vs. pilT^{K136A}	****	<0.0001	
ΔpilTΔpilU vs. pilT^{K136A} ΔpilU	ns	>0.9999	
ΔpilTΔpilU vs. pilT^{E204A}	ns	0.9904	
ΔpilTΔpilU vs. pilT^{E204A}/^{K136A}	****	<0.0001	
ΔpilTΔpilU vs. pilT^{E204A} ΔpilU	ns	>0.9999	
ΔpilTΔpilU vs. pilT^{E204A}/^{K136A} ΔpilU	ns	0.9718	
pilT^{K136A} vs. pilT^{K136A} ΔpilU	****	<0.0001	
pilT^{K136A} vs. pilT^{E204A}	****	<0.0001	
pilT^{K136A} vs. pilT$^{E204A/K136A}$	ns	0.9992	
pilT^{K136A} vs. pilT^{E204A} ΔpilU	****	<0.0001	
pilT^{K136A} vs. pilT^{E204A}/^{K136A} ΔpilU	****	<0.0001	
pilT^{K136A} ΔpilU vs. pilT^{E204A}	ns	0.9998	
pilT^{K136A} ΔpilU vs. pilT^{E204A}/^{K136A}	***	0.0001	
pilT^{K136A} ΔpilU vs. pilT^{E204A} ΔpilU	ns	0.9936	
pilT^{K136A} ΔpilU vs. pilT^{E204A}/^{K136A} ΔpilU	ns	0.8538	
pilT^{E204A} vs. pilT$^{E204A/K136A}$	***	0.0004	
pilT^{E204A} vs. pilT^{E204A} ΔpilU	ns	0.8779	
pilT^{E204A} vs. pilT^{E204A}/^{K136A} ΔpilU	ns	0.5286	
pilT^{E204A}/^{K136A} vs. pilT^{E204A} ΔpilU	****	<0.0001	
pilT^{E204A}/^{K136A} vs. pilT^{E204A}/^{K136A} ΔpilU	****	<0.0001	
pilT^{E204A} ΔpilU vs. pilT^{E204A}/^{K136A} ΔpilU	ns	0.9996	
**Figure 1F - Tukey's multiple comparison test**			
Parent vs. ΔpilT	****	<0.0001	# of families = 1
Parent vs. ΔpilU	****	<0.0001	# of comparisons per family = 6
Parent vs. ΔpilTΔpilU	****	<0.0001	Alpha = 0.05
ΔpilT vs. ΔpilU	ns	0.9956	
ΔpilT vs. ΔpilTΔpilU	ns	0.5137	
ΔpilU vs. ΔpilTΔpilU	ns	0.738	
**Figure 1H - Tukey's multiple comparison test**			
Parent vs. ΔpilT	****	<0.0001	# of families = 1
Parent vs. ΔpilU	****	<0.0001	# of comparisons per family = 6
Parent vs. ΔpilTΔpilU	****	<0.0001	Alpha = 0.05
ΔpilT vs. ΔpilU	**	0.0028	
ΔpilT vs. ΔpilTΔpilU	ns	0.9806	
ΔpilU vs. ΔpilTΔpilU	**	0.0051	
**Figure 2A - Tukey's multiple comparison test**			
Parent vs. pilU^{L199C}	ns	>0.9999	# of families = 1
Parent vs. pilT^{K136A} pilU^{L199C}	ns	0.9978	# of comparisons per family = 45
Parent vs. ΔpilT pilU^{L199C}	****	<0.0001	Alpha = 0.05

Parent vs. pilT^{L201C}	ns	>0.9999	
Parent vs. pilT^{L201C} ΔpilU	ns	0.4971	
Parent vs. pilT^{K136A}	ns	>0.9999	
Parent vs. pilU^{K134A}	ns	0.4696	
Parent vs. pilT^{K136A} pilU^{K134A}	****	<0.0001	
Parent vs. ΔpilU	ns	0.9608	
pilU^{L199C} vs. pilT^{K136A} pilU^{L199C}	ns	0.9864	
pilU^{L199C} vs. ΔpilT pilU^{L199C}	****	<0.0001	
pilU^{L199C} vs. pilT^{L201C}	ns	0.9999	
pilU^{L199C} vs. pilT^{L201C} ΔpilU	ns	0.4266	
pilU^{L199C} vs. pilT^{K136A}	ns	>0.9999	
pilU^{L199C} vs. pilU^{K134A}	ns	0.403	
pilU^{L199C} vs. pilT^{K136A} pilU^{K134A}	****	<0.0001	
pilU^{L199C} vs. ΔpilU	ns	0.9085	
pilT^{K136A} pilU^{L199C} vs. ΔpilT pilU^{L199C}	****	<0.0001	
pilT^{K136A} pilU^{L199C} vs. pilT^{L201C}	ns	>0.9999	
pilT^{K136A} pilU^{L199C} vs. pilT^{L201C} ΔpilU	ns	0.9608	
pilT^{K136A} pilU^{L199C} vs. pilT^{K136A}	ns	>0.9999	
pilT^{K136A} pilU^{L199C} vs. pilU^{K134A}	ns	0.9525	
pilT^{K136A} pilU^{L199C} vs. pilT^{K136A} pilU^{K134A}	****	<0.0001	
pilT^{K136A} pilU^{L199C} vs. ΔpilU	ns	>0.9999	
ΔpilT pilU^{L199C} vs. pilT^{L201C}	****	<0.0001	
ΔpilT pilU^{L199C} vs. pilT^{L201C} ΔpilU	****	<0.0001	
ΔpilT pilU^{L199C} vs. pilT^{K136A}	****	<0.0001	
ΔpilT pilU^{L199C} vs. pilU^{K134A}	****	<0.0001	
ΔpilT pilU^{L199C} vs. pilT^{K136A} pilU^{K134A}	****	<0.0001	
ΔpilT pilU^{L199C} vs. ΔpilU	****	<0.0001	
pilT^{L201C} vs. pilT^{L201C} ΔpilU	ns	0.7827	
pilT^{L201C} vs. pilT^{K136A}	ns	>0.9999	
pilT^{L201C} vs. pilU^{K134A}	ns	0.7604	
pilT^{L201C} vs. pilT^{K136A} pilU^{K134A}	****	<0.0001	
pilT^{L201C} vs. ΔpilU	ns	0.9968	
pilT^{L201C} ΔpilU vs. pilT^{K136A}	ns	0.7609	
pilT^{L201C} ΔpilU vs. pilU^{K134A}	ns	>0.9999	
pilT^{L201C} ΔpilU vs. pilT^{K136A} pilU^{K134A}	****	<0.0001	
pilT^{L201C} ΔpilU vs. ΔpilU	ns	0.9969	
pilT^{K136A} vs. pilU^{K134A}	ns	0.7378	
pilT^{K136A} vs. pilT^{K136A} pilU^{K134A}	****	<0.0001	
pilT^{K136A} vs. ΔpilU	ns	0.9956	
pilU^{K134A} vs. pilT^{K136A} pilU^{K134A}	****	<0.0001	
pilU^{K134A} vs. ΔpilU	ns	0.9957	
pilT^{K136A} pilU^{K134A} vs. ΔpilU	****	<0.0001	

Figure 2B - Tukey's multiple comparison test			
Parent vs. ΔpilU	****	<0.0001	# of families = 1
Parent vs. pilT^{K136A}	****	<0.0001	# of comparisons per family = 45
Parent vs. pilU^{K134A}	****	<0.0001	Alpha = 0.05
Parent vs. pilT^{K136A} pilU^{K134A}	****	<0.0001	
Parent vs. pilT^{K136A} pilU^{L199C}	****	<0.0001	
Parent vs. pilU^{L199C}	ns	0.6117	
Parent vs. pilT^{L201C}	ns	>0.9999	
Parent vs. pilT^{L201C} ΔpilU	****	<0.0001	
Parent vs. ΔpilT pilU^{L199C}	****	<0.0001	
ΔpilU vs. pilT^{K136A}	****	<0.0001	
ΔpilU vs. pilU^{K134A}	ns	0.1821	
ΔpilU vs. pilT^{K136A} pilU^{K134A}	****	<0.0001	
ΔpilU vs. pilT^{K136A} pilU^{L199C}	****	<0.0001	
ΔpilU vs. pilU^{L199C}	***	0.0003	
ΔpilU vs. pilT^{L201C}	****	<0.0001	
ΔpilU vs. pilT^{L201C} ΔpilU	*	0.014	
ΔpilU vs. ΔpilT pilU^{L199C}	****	<0.0001	
pilT^{K136A} vs. pilU^{K134A}	****	<0.0001	
pilT^{K136A} vs. pilT^{K136A} pilU^{K134A}	ns	0.3556	
pilT^{K136A} vs. pilT^{K136A} pilU^{L199C}	ns	0.9694	
pilT^{K136A} vs. pilU^{L199C}	****	<0.0001	
pilT^{K136A} vs. pilT^{L201C}	****	<0.0001	
pilT^{K136A} vs. pilT^{L201C} ΔpilU	****	<0.0001	
pilT^{K136A} vs. ΔpilT pilU^{L199C}	ns	0.1504	
pilU^{K134A} vs. pilT^{K136A} pilU^{K134A}	****	<0.0001	
pilU^{K134A} vs. pilT^{K136A} pilU^{L199C}	****	<0.0001	
pilU^{K134A} vs. pilU^{L199C}	****	<0.0001	
pilU^{K134A} vs. pilT^{L201C}	****	<0.0001	
pilU^{K134A} vs. pilT^{L201C} ΔpilU	ns	0.9841	
pilU^{K134A} vs. ΔpilT pilU^{L199C}	****	<0.0001	
pilT^{K136A} pilU^{K134A} vs. pilT^{K136A} pilU^{L199C}	ns	0.8001	
pilT^{K136A} pilU^{K134A} vs. pilU^{L199C}	****	<0.0001	
pilT^{K136A} pilU^{K134A} vs. pilT^{L201C}	****	<0.0001	
pilT^{K136A} pilU^{K134A} vs. pilT^{L201C} ΔpilU	****	<0.0001	
pilT^{K136A} pilU^{K134A} vs. ΔpilT pilU^{L199C}	ns	>0.9999	
pilT^{K136A} pilU^{L199C} vs. pilU^{L199C}	****	<0.0001	
pilT^{K136A} pilU^{L199C} vs. pilT^{L201C}	****	<0.0001	
pilT^{K136A} pilU^{L199C} vs. pilT^{L201C} ΔpilU	****	<0.0001	
pilT^{K136A} pilU^{L199C} vs. ΔpilT pilU^{L199C}	ns	0.6067	
pilU^{L199C} vs. pilT^{L201C}	ns	0.7303	

pilU^{L199C} vs. pilT^{L201C} ΔpilU	****	<0.0001	
pilU^{L199C} vs. ΔpilT pilU^{L199C}	****	<0.0001	
pilT^{L201C} vs. pilT^{L201C} ΔpilU	****	<0.0001	
pilT^{L201C} vs. ΔpilT pilU^{L199C}	****	<0.0001	
pilT^{L201C} ΔpilU vs. ΔpilT pilU^{L199C}	****	<0.0001	
**Figure 4C - Tukey's multiple comparison test**			
Parent vs. ΔpilU	ns	>0.9999	# of families = 1
Parent vs. ΔpilT	****	<0.0001	# of comparisons per family = 36
Parent vs. pilT^{K136A}	ns	0.9998	Alpha = 0.05
Parent vs. Ptac-pilT^{K136A}	ns	0.9537	
Parent vs. Ptac-pilT^{K136A} ΔpilU	****	<0.0001	
Parent vs. Ptac-pilU^{K134A}	ns	0.9996	
Parent vs. Ptac-pilU^{K134A} pilT^{K136A}	****	<0.0001	
Parent vs. Ptac-pilT^{K136A} Ptac-pilU^{K134A}	****	<0.0001	
ΔpilU vs. ΔpilT	****	<0.0001	
ΔpilU vs. pilT^{K136A}	ns	>0.9999	
ΔpilU vs. Ptac-pilT^{K136A}	ns	0.9844	
ΔpilU vs. Ptac-pilT^{K136A} ΔpilU	****	<0.0001	
ΔpilU vs. Ptac-pilU^{K134A}	ns	>0.9999	
ΔpilU vs. Ptac-pilU^{K134A} pilT^{K136A}	****	<0.0001	
ΔpilU vs. Ptac-pilT^{K136A} Ptac-pilU^{K134A}	****	<0.0001	
ΔpilT vs. pilT^{K136A}	****	<0.0001	
ΔpilT vs. Ptac-pilT^{K136A}	****	<0.0001	
ΔpilT vs. Ptac-pilT^{K136A} ΔpilU	ns	0.9408	
ΔpilT vs. Ptac-pilU^{K134A}	****	<0.0001	
ΔpilT vs. Ptac-pilU^{K134A} pilT^{K136A}	ns	0.3939	
ΔpilT vs. Ptac-pilT^{K136A} Ptac-pilU^{K134A}	ns	0.542	
pilT^{K136A} vs. Ptac-pilT^{K136A}	ns	0.9988	
pilT^{K136A} vs. Ptac-pilT^{K136A} ΔpilU	****	<0.0001	
pilT^{K136A} vs. Ptac-pilU^{K134A}	ns	>0.9999	
pilT^{K136A} vs. Ptac-pilU^{K134A} pilT^{K136A}	****	<0.0001	
pilT^{K136A} vs. Ptac-pilT^{K136A} Ptac-pilU^{K134A}	****	<0.0001	
Ptac-pilT^{K136A} vs. Ptac-pilT^{K136A} ΔpilU	****	<0.0001	
Ptac-pilT^{K136A} vs. Ptac-pilU^{K134A}	ns	0.9994	
Ptac-pilT^{K136A} vs. Ptac-pilU^{K134A} pilT^{K136A}	****	<0.0001	
Ptac-pilT^{K136A} vs. Ptac-pilT^{K136A} Ptac-pilU^{K134A}	****	<0.0001	
Ptac-pilT^{K136A} ΔpilU vs. Ptac-pilU^{K134A}	****	<0.0001	
Ptac-pilT^{K136A} ΔpilU vs. Ptac-pilU^{K134A} pilT^{K136A}	ns	0.9789	
Ptac-pilT^{K136A} ΔpilU vs. Ptac-pilT^{K136A} Ptac-pilU^{K134A}	ns	0.9964	
Ptac-pilU^{K134A} vs. Ptac-pilU^{K134A} pilT^{K136A}	****	<0.0001	
Ptac-pilU^{K134A} vs. Ptac-pilT^{K136A} Ptac-pilU^{K134A}	****	<0.0001	

Ptac-pilU^{K134A} pilT^{K136A} vs. Ptac-pilT^{K136A} Ptac-pilU^{K134A}	ns	>0.9999	
**Figure 5 - Tukey's multiple comparison test**			
pilT^{K136A} ΔpilU vs. pilT^{K136A} pilU^{K137A}	ns	0.9546	# of families = 1
pilT^{K136A} ΔpilU vs. pilT^{K136A}	****	<0.0001	# of comparisons per family = 36
pilT^{K136A} ΔpilU vs. pilU^{K137A}	****	<0.0001	Alpha = 0.05
pilT^{K136A} ΔpilU vs. Parent	****	<0.0001	
pilT^{K136A} ΔpilU vs. ΔpilT	ns	>0.9999	
pilT^{K136A} ΔpilU vs. ΔpilU	****	<0.0001	
pilT^{K136A} ΔpilU vs. ΔpilTU	ns	>0.9999	
pilT^{K136A} ΔpilU vs. ΔcomP	ns	0.7185	
pilT^{K136A} pilU^{K137A} vs. pilT^{K136A}	****	<0.0001	
pilT^{K136A} pilU^{K137A} vs. pilU^{K137A}	****	<0.0001	
pilT^{K136A} pilU^{K137A} vs. Parent	****	<0.0001	
pilT^{K136A} pilU^{K137A} vs. ΔpilT	ns	0.9681	
pilT^{K136A} pilU^{K137A} vs. ΔpilU	****	<0.0001	
pilT^{K136A} pilU^{K137A} vs. ΔpilTU	ns	0.9694	
pilT^{K136A} pilU^{K137A} vs. ΔcomP	ns	>0.9999	
pilT^{K136A} vs. pilU^{K137A}	**	0.0082	
pilT^{K136A} vs. Parent	****	<0.0001	
pilT^{K136A} vs. ΔpilT	****	<0.0001	
pilT^{K136A} vs. ΔpilU	***	0.0003	
pilT^{K136A} vs. ΔpilTU	****	<0.0001	
pilT^{K136A} vs. ΔcomP	****	<0.0001	
pilU^{K137A} vs. Parent	****	<0.0001	
pilU^{K137A} vs. ΔpilT	****	<0.0001	
pilU^{K137A} vs. ΔpilU	ns	0.9315	
pilU^{K137A} vs. ΔpilTU	****	<0.0001	
pilU^{K137A} vs. ΔcomP	****	<0.0001	
Parent vs. ΔpilT	****	<0.0001	
Parent vs. ΔpilU	****	<0.0001	
Parent vs. ΔpilTU	****	<0.0001	
Parent vs. ΔcomP	****	<0.0001	
ΔpilT vs. ΔpilU	****	<0.0001	
ΔpilT vs. ΔpilTU	ns	>0.9999	
ΔpilT vs. ΔcomP	ns	0.7633	
ΔpilU vs. ΔpilTU	****	<0.0001	
ΔpilU vs. ΔcomP	****	<0.0001	
ΔpilTU vs. ΔcomP	ns	0.7681	
**Supplemental Figure 1 - Dunnett's multiple comparison test**			
Parent Ptac-PilT vs. ΔpilT ΔpilU Ptac-PilT	ns	0.9999	# of families = 2
Parent Ptac-PilT vs. pilT^{K136A} ΔpilU Ptac-PilT	ns	0.9999	# of comparisons per family = 6

Parent Ptac-PilT vs. pilT^{K136A} pilU^{K134A} Ptac-PilT	ns	0.37	Alpha = 0.05
Parent Ptac-PilT vs. ΔpilT Ptac-PilT	ns	0.9999	
Parent Ptac-PilT vs. pilT$^{K136A/E204A}$ ΔpilU Ptac-PilT	ns	0.9996	
Parent Ptac-PilT vs. pilT^{E204A} ΔpilU Ptac-PilT	ns	0.9779	
Parent Ptac-PilU vs. ΔpilT ΔpilU Ptac-PilU	****	0.0001	
Parent Ptac-PilU vs. pilT^{K136A} ΔpilU Ptac-PilU	ns	0.8282	
Parent Ptac-PilU vs. pilT^{K136A} pilU^{K134A} Ptac-PilU	ns	0.3199	
Parent Ptac-PilU vs. ΔpilT Ptac-PilU	****	0.0001	
Parent Ptac-PilU vs. pilT$^{K136A/E204A}$ ΔpilU Ptac-PilU	ns	0.0732	
Parent Ptac-PilU vs. pilT^{E204A} ΔpilU Ptac-PilU	ns	0.9999	
**Supplemental Figure 2 (T18 x T25) - Dunnett's multiple comparison test**			
vector x PilT vs. PilT x PilT	****	<0.0001	# of families = 6
vector x PilT vs. PilU x PilT	*	0.0343	# of comparisons per family = 6
vector x PilT vs. PilT^{K136A} x PilT	****	<0.0001	Alpha = 0.05
vector x PilT vs. PilU^{K134A} x PilT	**	0.0043	
vector x PilT vs. PilC x PilT	***	0.0002	
vector x PilU vs. PilT x PilU	*	0.0464	
vector x PilU vs. PilU x PilU	**	0.0016	
vector x PilU vs. PilT^{K136A} x PilU	ns	0.2389	
vector x PilU vs. PilU^{K134A} x PilU	***	0.0005	
vector x PilU vs. PilC x PilU	ns	0.0849	
vector x PilT^{K136A} vs. PilT x PilT^{K136A}	**	0.0031	
vector x PilT^{K136A} vs. PilU x PilT^{K136A}	ns	0.9997	
vector x PilT^{K136A} vs. PilT^{K136A} x PilT^{K136A}	ns	0.1368	
vector x PilT^{K136A} vs. PilU^{K134A} x PilT^{K136A}	ns	0.9984	
vector x PilT^{K136A} vs. PilC x PilT^{K136A}	*	0.0349	
vector x PilU^{K134A} vs. PilT x PilU^{K134A}	ns	0.1185	
vector x PilU^{K134A} vs. PilU x PilU^{K134A}	***	0.0004	
vector x PilU^{K134A} vs. PilT^{K136A} x PilU^{K134A}	ns	0.4273	
vector x PilU^{K134A} vs. PilU^{K134A} x PilU^{K134A}	**	0.003	
vector x PilU^{K134A} vs. PilC x PilU^{K134A}	*	0.013	
vector x PilC vs. PilT x PilC	ns	0.0724	
vector x PilC vs. PilU x PilC	ns	0.4038	
vector x PilC vs. PilT^{K136A} x PilC	ns	0.1408	
vector x PilC vs. PilU^{K134A} x PilC	ns	0.1544	
vector x PilC vs. PilC x PilC	****	0.0001	
vector x vector vs. PilT x vector	ns	0.9999	
vector x vector vs. PilU x vector	ns	0.9999	
vector x vector vs. PilT^{K136A} x vector	ns	0.9999	
vector x vector vs. PilU^{K134A} x vector	ns	0.9999	
vector x vector vs. PilC x vector	ns	0.9999	

Supplemental Figure 3A - Tukey's multiple comparison test			
Parent vs. ΔpilT	***	0.0002	# of families = 1
Parent vs. pilT^{K136A}, 6xHis-pilU	ns	>0.9999	# of comparisons per family = 36
Parent vs. 6xHis-pilT	ns	0.9508	Alpha = 0.05
Parent vs. 6xHis-pilU	ns	0.9235	
Parent vs. pilT^{K136A}, 3xFLAG-pilU	ns	0.9553	
Parent vs. 3xFLAG-pilT	ns	>0.9999	
Parent vs. 3xFLAG-pilU	ns	0.9949	
Parent vs. pilT^{K136A}	ns	0.9865	
ΔpilT vs. pilT^{K136A}, 6xHis-pilU	***	0.0007	
ΔpilT vs. 6xHis-pilT	*	0.0209	
ΔpilT vs. 6xHis-pilU	*	0.0261	
ΔpilT vs. pilT^{K136A}, 3xFLAG-pilU	*	0.0201	
ΔpilT vs. 3xFLAG-pilT	**	0.0014	
ΔpilT vs. 3xFLAG-pilU	**	0.0093	
ΔpilT vs. pilT^{K136A}	*	0.0126	
pilT^{K136A}, 6xHis-pilU vs. 6xHis-pilT	ns	0.9279	
pilT^{K136A}, 6xHis-pilU vs. 6xHis-pilU	ns	0.8986	
pilT^{K136A}, 6xHis-pilU vs. pilT^{K136A}, 3xFLAG-pilU	ns	0.9329	
pilT^{K136A}, 6xHis-pilU vs. 3xFLAG-pilT	ns	>0.9999	
pilT^{K136A}, 6xHis-pilU vs. 3xFLAG-pilU	ns	0.9862	
pilT^{K136A}, 6xHis-pilU vs. pilT^{K136A}	ns	0.9724	
6xHis-pilT vs. 6xHis-pilU	ns	>0.9999	
6xHis-pilT vs. pilT^{K136A}, 3xFLAG-pilU	ns	>0.9999	
6xHis-pilT vs. 3xFLAG-pilT	ns	0.98	
6xHis-pilT vs. 3xFLAG-pilU	ns	>0.9999	
6xHis-pilT vs. pilT^{K136A}	ns	>0.9999	
6xHis-pilU vs. pilT^{K136A}, 3xFLAG-pilU	ns	>0.9999	
6xHis-pilU vs. 3xFLAG-pilT	ns	0.9671	
6xHis-pilU vs. 3xFLAG-pilU	ns	>0.9999	
6xHis-pilU vs. pilT^{K136A}	ns	>0.9999	
pilT^{K136A}, 3xFLAG-pilU vs. 3xFLAG-pilT	ns	0.982	
pilT^{K136A}, 3xFLAG-pilU vs. 3xFLAG-pilU	ns	>0.9999	
pilT^{K136A}, 3xFLAG-pilU vs. pilT^{K136A}	ns	>0.9999	
3xFLAG-pilT vs. 3xFLAG-pilU	ns	0.9983	
3xFLAG-pilT vs. pilT^{K136A}	ns	0.9951	
3xFLAG-pilU vs. pilT^{K136A}	ns	>0.9999	
**Supplemental Figure 3B - Tukey's multiple comparison test**			
Parent vs. ΔpilT	ns	0.2194	# of families = 1
Parent vs. Ptac-3xFLAG-pilT	ns	>0.9999	# of comparisons per family = 66
Parent vs. Ptac-3xFLAG-pilT ΔpilT	ns	0.9989	Alpha = 0.05
Parent vs. Ptac-3xFLAG-pilU	ns	0.9995	
Parent vs. Ptac-3xFLAG-pilU, pilT^{K136A} ΔpilU	ns	0.9127	

Parent vs. Ptac-3xFLAG-pilT^{K136A}	ns	0.9505	
Parent vs. Ptac-3xFLAG-pilU^{K134A}	ns	0.9999	
Parent vs. Ptac-3xFLAG-pilT^{K136A}, ΔpilU	ns	0.2197	
Parent vs. Ptac-3xFLAG-pilU^{K134A}, pilT^{K136A}	ns	0.2199	
Parent vs. ΔpilU	ns	0.998	
Parent vs. pilT^{K136A}	ns	0.9912	
ΔpilT vs. Ptac-3xFLAG-pilT	ns	0.0832	
ΔpilT vs. Ptac-3xFLAG-pilT ΔpilT	*	0.0472	
ΔpilT vs. Ptac-3xFLAG-pilU	ns	0.5898	
ΔpilT vs. Ptac-3xFLAG-pilU, pilT^{K136A} ΔpilU	ns	0.9879	
ΔpilT vs. Ptac-3xFLAG-pilT^{K136A}	*	0.0123	
ΔpilT vs. Ptac-3xFLAG-pilU^{K134A}	ns	0.0697	
ΔpilT vs. Ptac-3xFLAG-pilT^{K136A}, ΔpilU	ns	>0.9999	
ΔpilT vs. Ptac-3xFLAG-pilU^{K134A}, pilT^{K136A}	ns	>0.9999	
ΔpilT vs. ΔpilU	ns	0.8312	
ΔpilT vs. pilT^{K136A}	ns	0.9042	
Ptac-3xFLAG-pilT vs. Ptac-3xFLAG-pilT ΔpilT	ns	>0.9999	
Ptac-3xFLAG-pilT vs. Ptac-3xFLAG-pilU	ns	0.9531	
Ptac-3xFLAG-pilT vs. Ptac-3xFLAG-pilU, pilT^{K136A} ΔpilU	ns	0.6364	
Ptac-3xFLAG-pilT vs. Ptac-3xFLAG-pilT^{K136A}	ns	0.9998	
Ptac-3xFLAG-pilT vs. Ptac-3xFLAG-pilU^{K134A}	ns	>0.9999	
Ptac-3xFLAG-pilT vs. Ptac-3xFLAG-pilT^{K136A}, ΔpilU	ns	0.0833	
Ptac-3xFLAG-pilT vs. Ptac-3xFLAG-pilU^{K134A}, pilT^{K136A}	ns	0.0834	
Ptac-3xFLAG-pilT vs. ΔpilU	ns	0.9305	
Ptac-3xFLAG-pilT vs. pilT^{K136A}	ns	0.8688	
Ptac-3xFLAG-pilT ΔpilT vs. Ptac-3xFLAG-pilU	ns	0.8682	
Ptac-3xFLAG-pilT ΔpilT vs. Ptac-3xFLAG-pilU, pilT^{K136A} ΔpilU	ns	0.4801	
Ptac-3xFLAG-pilT ΔpilT vs. Ptac-3xFLAG-pilT^{K136A}	ns	>0.9999	
Ptac-3xFLAG-pilT ΔpilT vs. Ptac-3xFLAG-pilU^{K134A}	ns	>0.9999	
Ptac-3xFLAG-pilT ΔpilT vs. Ptac-3xFLAG-pilT^{K136A}, ΔpilU	*	0.0473	
Ptac-3xFLAG-pilT ΔpilT vs. Ptac-3xFLAG-pilU^{K134A}, pilT^{K136A}	*	0.0473	
Ptac-3xFLAG-pilT ΔpilT vs. ΔpilU	ns	0.8381	
Ptac-3xFLAG-pilT ΔpilT vs. pilT^{K136A}	ns	0.7454	
Ptac-3xFLAG-pilU vs. Ptac-3xFLAG-pilU, pilT^{K136A} ΔpilU	ns	0.9991	
Ptac-3xFLAG-pilU vs. Ptac-3xFLAG-pilT^{K136A}	ns	0.5332	
Ptac-3xFLAG-pilU vs. Ptac-3xFLAG-pilU^{K134A}	ns	0.9319	
Ptac-3xFLAG-pilU vs. Ptac-3xFLAG-pilT^{K136A}, ΔpilU	ns	0.5902	

Ptac-3xFLAG-pilU vs. Ptac-3xFLAG-pilU^{K134A}, pilT^{K136A}	ns	0.5906	
Ptac-3xFLAG-pilU vs. ΔpilU	ns	>0.9999	
Ptac-3xFLAG-pilU vs. pilT^{K136A}	ns	>0.9999	
Ptac-3xFLAG-pilU, pilT^{K136A} ΔpilU vs. Ptac-3xFLAG-pilT^{K136A}	ns	0.2052	
Ptac-3xFLAG-pilU, pilT^{K136A} ΔpilU vs. Ptac-3xFLAG-pilU^{K134A}	ns	0.5864	
Ptac-3xFLAG-pilU, pilT^{K136A} ΔpilU vs. Ptac-3xFLAG-pilT^{K136A}, ΔpilU	ns	0.988	
Ptac-3xFLAG-pilU, pilT^{K136A} ΔpilU vs. Ptac-3xFLAG-pilU^{K134A}, pilT^{K136A}	ns	0.988	
Ptac-3xFLAG-pilU, pilT^{K136A} ΔpilU vs. ΔpilU	ns	>0.9999	
Ptac-3xFLAG-pilU, pilT^{K136A} ΔpilU vs. pilT^{K136A}	ns	>0.9999	
Ptac-3xFLAG-pilT^{K136A} vs. Ptac-3xFLAG-pilU^{K134A}	ns	>0.9999	
Ptac-3xFLAG-pilT^{K136A} vs. Ptac-3xFLAG-pilT^{K136A}, ΔpilU	*	0.0123	
Ptac-3xFLAG-pilT^{K136A} vs. Ptac-3xFLAG-pilU^{K134A}, pilT^{K136A}	*	0.0123	
Ptac-3xFLAG-pilT^{K136A} vs. ΔpilU	ns	0.5225	
Ptac-3xFLAG-pilT^{K136A} vs. pilT^{K136A}	ns	0.4139	
Ptac-3xFLAG-pilU^{K134A} vs. Ptac-3xFLAG-pilT^{K136A}, ΔpilU	ns	0.0698	
Ptac-3xFLAG-pilU^{K134A} vs. Ptac-3xFLAG-pilU^{K134A}, pilT^{K136A}	ns	0.0699	
Ptac-3xFLAG-pilU^{K134A} vs. ΔpilU	ns	0.906	
Ptac-3xFLAG-pilU^{K134A} vs. pilT^{K136A}	ns	0.8338	
Ptac-3xFLAG-pilT^{K136A}, ΔpilU vs. Ptac-3xFLAG-pilU^{K134A}, pilT^{K136A}	ns	>0.9999	
Ptac-3xFLAG-pilT^{K136A}, ΔpilU vs. ΔpilU	ns	0.8315	
Ptac-3xFLAG-pilT^{K136A}, ΔpilU vs. pilT^{K136A}	ns	0.9044	
Ptac-3xFLAG-pilU^{K134A}, pilT^{K136A} vs. ΔpilU	ns	0.8318	
Ptac-3xFLAG-pilU^{K134A}, pilT^{K136A} vs. pilT^{K136A}	ns	0.9046	
ΔpilU vs. pilT^{K136A}	ns	>0.9999	
**Supplemental Figure 3D - Tukey's multiple comparison test**			
Parent vs. ΔpilT ΔpilU	****	<0.0001	# of families = 1
Parent vs. pilU^{K134A}	ns	0.9994	# of comparisons per family = 55
Parent vs. pilT^{K136A} ΔpilU	****	<0.0001	Alpha = 0.05
Parent vs. pilT^{K136A} pilU^{K134A}	****	<0.0001	
Parent vs. P_{tac}-pilT	ns	0.999	
Parent vs. ΔpilT Ptac-pilT	ns	>0.9999	
Parent vs. P_{tac}-pilU	ns	0.999	
Parent vs. pilT^{K136A} ΔpilU Ptac-pilU	ns	0.9481	
Parent vs. ΔpilA	****	<0.0001	

Parent vs. pilT^{K136A}	ns	>0.9999	
ΔpilT ΔpilU vs. pilU^{K134A}	****	<0.0001	
ΔpilT ΔpilU vs. pilT^{K136A} ΔpilU	ns	0.4878	
ΔpilT ΔpilU vs. pilT^{K136A} pilU^{K134A}	ns	>0.9999	
ΔpilT ΔpilU vs. Ptac-pilT	****	<0.0001	
ΔpilT ΔpilU vs. ΔpilT Ptac-pilT	****	<0.0001	
ΔpilT ΔpilU vs. Ptac-pilU	****	<0.0001	
ΔpilT ΔpilU vs. pilT^{K136A} ΔpilU Ptac-pilU	****	<0.0001	
ΔpilT ΔpilU vs. ΔpilA	**	0.0028	
ΔpilT ΔpilU vs. pilT^{K136A}	****	<0.0001	
pilU^{K134A} vs. pilT^{K136A} ΔpilU	****	<0.0001	
pilU^{K134A} vs. pilT^{K136A} pilU^{K134A}	****	<0.0001	
pilU^{K134A} vs. P$_{tac}$-pilT	ns	>0.9999	
pilU^{K134A} vs. ΔpilT Ptac-pilT	ns	>0.9999	
pilU^{K134A} vs. P$_{tac}$-pilU	ns	>0.9999	
pilU^{K134A} vs. pilT^{K136A} ΔpilU Ptac-pilU	ns	0.6391	
pilU^{K134A} vs. ΔpilA	****	<0.0001	
pilU^{K134A} vs. pilT^{K136A}	ns	0.9972	
pilT^{K136A} ΔpilU vs. pilT^{K136A} pilU^{K134A}	ns	0.6518	
pilT^{K136A} ΔpilU vs. Ptac-pilT	****	<0.0001	
pilT^{K136A} ΔpilU vs. ΔpilT Ptac-pilT	****	<0.0001	
pilT^{K136A} ΔpilU vs. Ptac-pilU	****	<0.0001	
pilT^{K136A} ΔpilU vs. pilT^{K136A} ΔpilU Ptac-pilU	****	<0.0001	
pilT^{K136A} ΔpilU vs. ΔpilA	ns	0.4692	
pilT^{K136A} ΔpilU vs. pilT^{K136A}	****	<0.0001	
pilT^{K136A} pilU^{K134A} vs. P$_{tac}$-pilT	****	<0.0001	
pilT^{K136A} pilU^{K134A} vs. ΔpilT Ptac-pilT	****	<0.0001	
pilT^{K136A} pilU^{K134A} vs. P$_{tac}$-pilU	****	<0.0001	
pilT^{K136A} pilU^{K134A} vs. pilT^{K136A} ΔpilU Ptac-pilU	****	<0.0001	
pilT^{K136A} pilU^{K134A} vs. ΔpilA	**	0.0057	
pilT^{K136A} pilU^{K134A} vs. pilT^{K136A}	****	<0.0001	
Ptac-pilT vs. ΔpilT Ptac-pilT	ns	>0.9999	
P$_{tac}$-pilT vs. P$_{tac}$-pilU	ns	>0.9999	
Ptac-pilT vs. pilT^{K136A} ΔpilU Ptac-pilU	ns	0.6089	
Ptac-pilT vs. ΔpilA	****	<0.0001	
P$_{tac}$-pilT vs. pilT^{K136A}	ns	0.9957	
ΔpilT Ptac-pilT vs. Ptac-pilU	ns	>0.9999	
ΔpilT Ptac-pilT vs. pilT^{K136A} ΔpilU Ptac-pilU	ns	0.7364	
ΔpilT Ptac-pilT vs. ΔpilA	****	<0.0001	
ΔpilT Ptac-pilT vs. pilT^{K136A}	ns	0.9995	
Ptac-pilU vs. pilT^{K136A} ΔpilU Ptac-pilU	ns	0.6068	
Ptac-pilU vs. ΔpilA	****	<0.0001	

P_{tac}-pilU vs. $pilT^{K136A}$	ns	0.9956	
$pilT^{K136A}$ ΔpilU Ptac-pilU vs. ΔpilA	****	<0.0001	
$pilT^{K136A}$ ΔpilU Ptac-pilU vs. $pilT^{K136A}$	ns	0.9772	
ΔpilA vs. $pilT^{K136A}$	****	<0.0001	
**Supplemental Figure 4 - Dunnett's multiple comparison test**			
Parent pmmB-pilT vs. ΔpilTU pmmB-pilT	****	0.0001	# of families = 2
Parent pmmB-pilT vs. ΔpilT pmmB-pilT	ns	0.8827	# of comparisons per family = 8
Parent pmmB-pilT vs. ΔpilU pmmB-pilT	****	0.0001	Alpha = 0.05
Parent pmmB-pilT vs. $pilT^{K136A}$ pmmB-pilT	ns	0.8666	
Parent pmmB-pilT vs. $pilT^{K136A}$ ΔpilU pmmB-pilT	****	0.0001	
Parent pmmB-pilT vs. $pilU^{K137A}$ pmmB-pilT	****	0.0001	
Parent pmmB-pilT vs. $pilT^{K136A}$ $pilU^{K137A}$ pmmB-pilT	****	0.0001	
Parent pmmB-pilU vs. ΔpilTU pmmB-pilU	Yes	****	
Parent pmmB-pilU vs. ΔpilT pmmB-pilU	Yes	****	
Parent pmmB-pilU vs. ΔpilU pmmB-pilU	No	ns	
Parent pmmB-pilU vs. $pilT^{K136A}$ pmmB-pilU	Yes	****	
Parent pmmB-pilU vs. $pilT^{K136A}$ ΔpilU pmmB-pilU	Yes	****	
Parent pmmB-pilU vs. $pilU^{K137A}$ pmmB-pilU	Yes	*	
Parent pmmB-pilU vs. $pilT^{K136A}$ $pilU^{K137A}$ pmmB-pilU	Yes	****	
Parent pmmB-pilU vs. Parent pmmB-pilU	No	ns	
**Supplemental Figure 5 - Tukey's multiple comparison test**			
Parent vs. ΔMSHA ΔTCP	ns	0.8726	# of families = 1
Parent vs. ΔpilTU	****	<0.0001	# of comparisons per family = 6
Parent vs. ΔpilTU ΔMSHA ΔTCP	****	<0.0001	Alpha = 0.05
ΔMSHA ΔTCP vs. ΔpilTU	****	<0.0001	
ΔMSHA ΔTCP vs. ΔpilTU ΔMSHA ΔTCP	****	<0.0001	
ΔpilTU vs. ΔpilTU ΔMSHA ΔTCP	ns	0.0927	

[#]Statistical differences were assessed by one-way ANOVA tests followed by a multiple comparisons post test (either Tukey's or Dunnett's) using GraphPad Prism software. For all transformation frequency experiments and frequency of pilus retraction experiments, statistical analyses were performed on the log transformed data (i.e. **Figs 1A, 1C, 2B, 4C** and **S1, S3A, S3B, S3D, S4, S5 Figs**).
